# Supplementary figures and images for: Attractive serial dependence overcomes repulsive neuronal adaptation
Source: PLoS Biol. 2022 Sep 6;20(9):e3001711. doi: 10.1371/journal.pbio.3001711 (PMC9447932; doi:10.1371/journal.pbio.3001711)

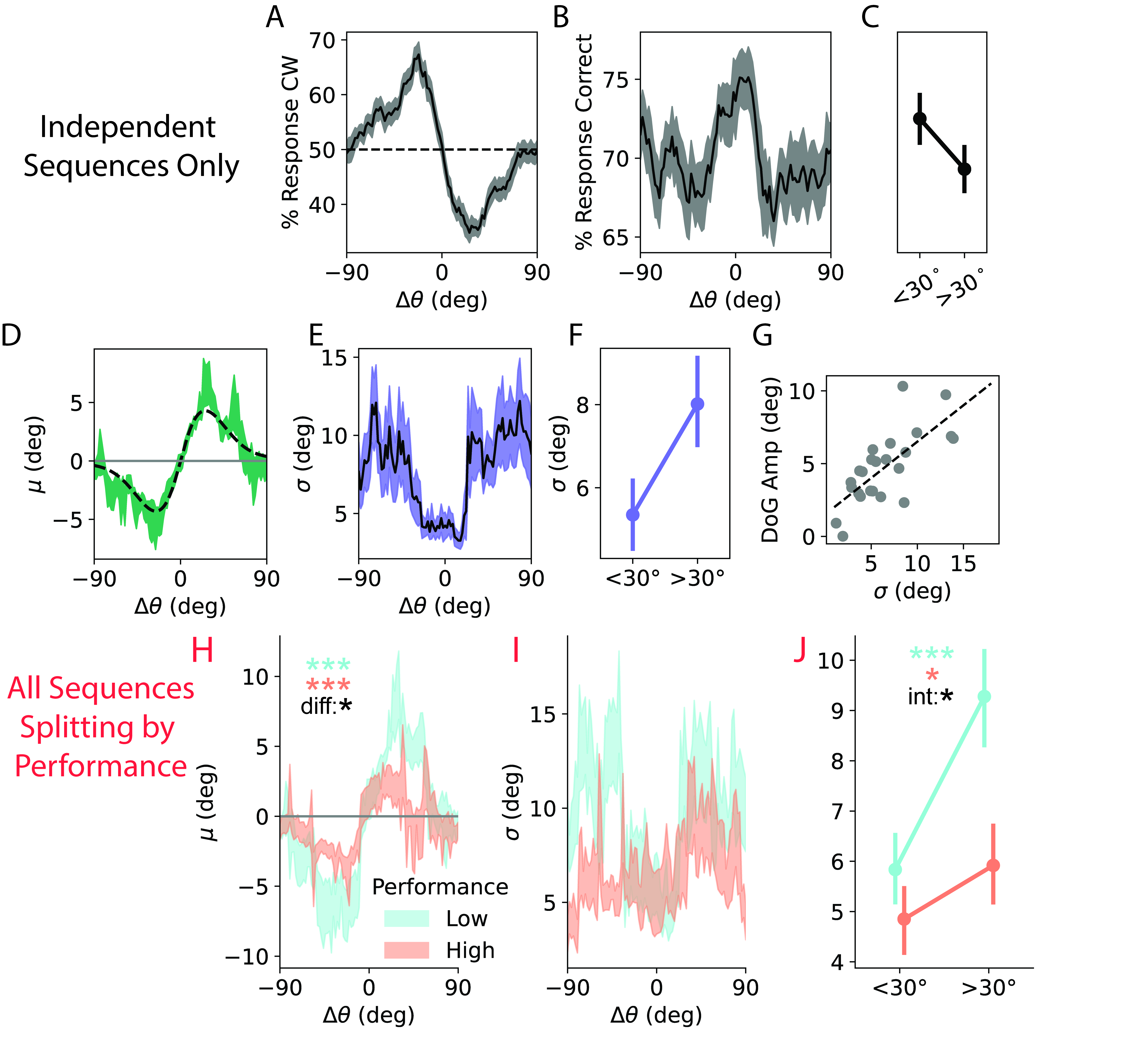

Supplement: S2 Fig — To confirm this manipulation did not drive any of our results, we repeated our behavioral analyses excluding participants with nonindependent sequences leaving a cohort of n = 25 with an average accuracy of 70.46 ± 1.14° at an average δθ of 4.97 ± 0.35°. (A, D) This cohort still showed significant serial dependence (DoG amp = 4.71 ± 0.49, t(23) = 9.4, p = 2.4*10−9; width 0.027 ± 0.0019, FWHM 43.68 ± 1.86°, (B, C) and had responses that were more accurate (t(24) = 3.14, p = 0.0023, (E, F) and precise following “close” stimuli (t(24) = −3.54, p = 0.0009, (G) Last, bias and variance were still positively correlated across this cohort (r(22) = 0.72, p = 0.00003, (H–J) Stimulus history effects are larger for worse performing participants. H: Serial dependence was significantly greater for less precise participants t(45) = −2.5, p = 0.012, unpaired t test comparing DoG Amplitude). (I–J) Variance was modulated significantly by stimulus history (low-performing: t(23) = 3.9 p = 0.0007; high-performing t(22) = 2.4, p = 0.02, 1-sample t tests), with a significant interaction between overall performance and the effect size (p = 0.017, mixed effects linear model). Data and code supporting this figure found here: https://osf.io/e5xw8/?view_only=e7c1da85aa684cc8830aec8d74afdcb4. DoG, Derivative of Gaussian; FWHM, full width at half maximum. (TIF) [file pbio.3001711.s002.tif]

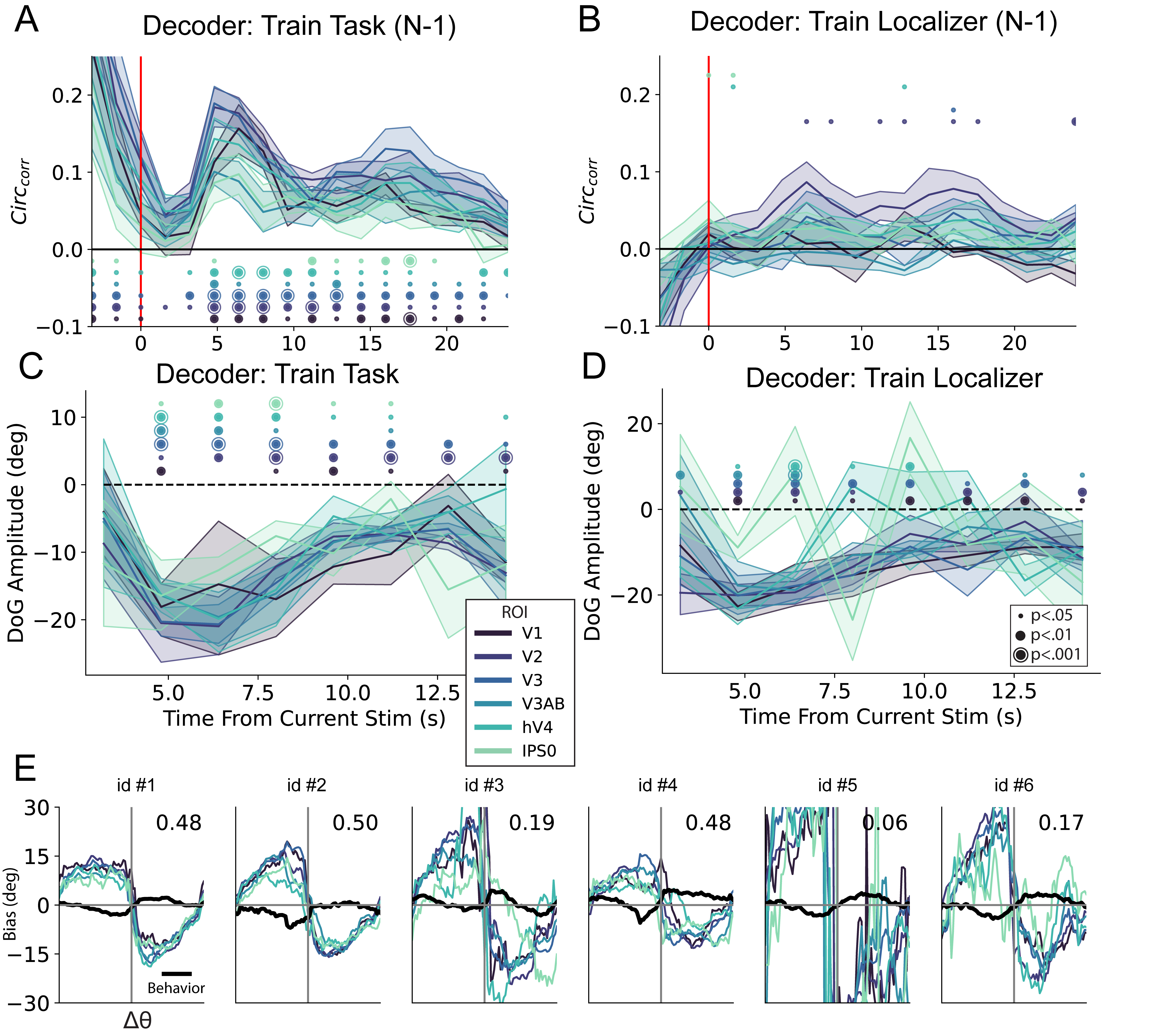

Supplement: S4 Fig — (A) Decoding of the previous stimulus dropped to chance around stimulus presentation before rebounding. (B) Decoding using sensory localizer data was consistently at chance during N+1 trial suggesting information relating to past stimulus is not stored in a sensory code. (C, D) Decoded biases across time for both decoders are consistently repulsive. (E) Bias curves for individual participants using the memory decoder across ROIs (see legend) overlayed with behavioral biases (black). Neural and behavioral biases are consistently in opposite directions. Note that id#3 exhibits peripheral repulsion. Data and code supporting this figure found here: https://osf.io/e5xw8/?view_only=e7c1da85aa684cc8830aec8d74afdcb4. ROI, region of interest. (TIF) [file pbio.3001711.s004.tif]

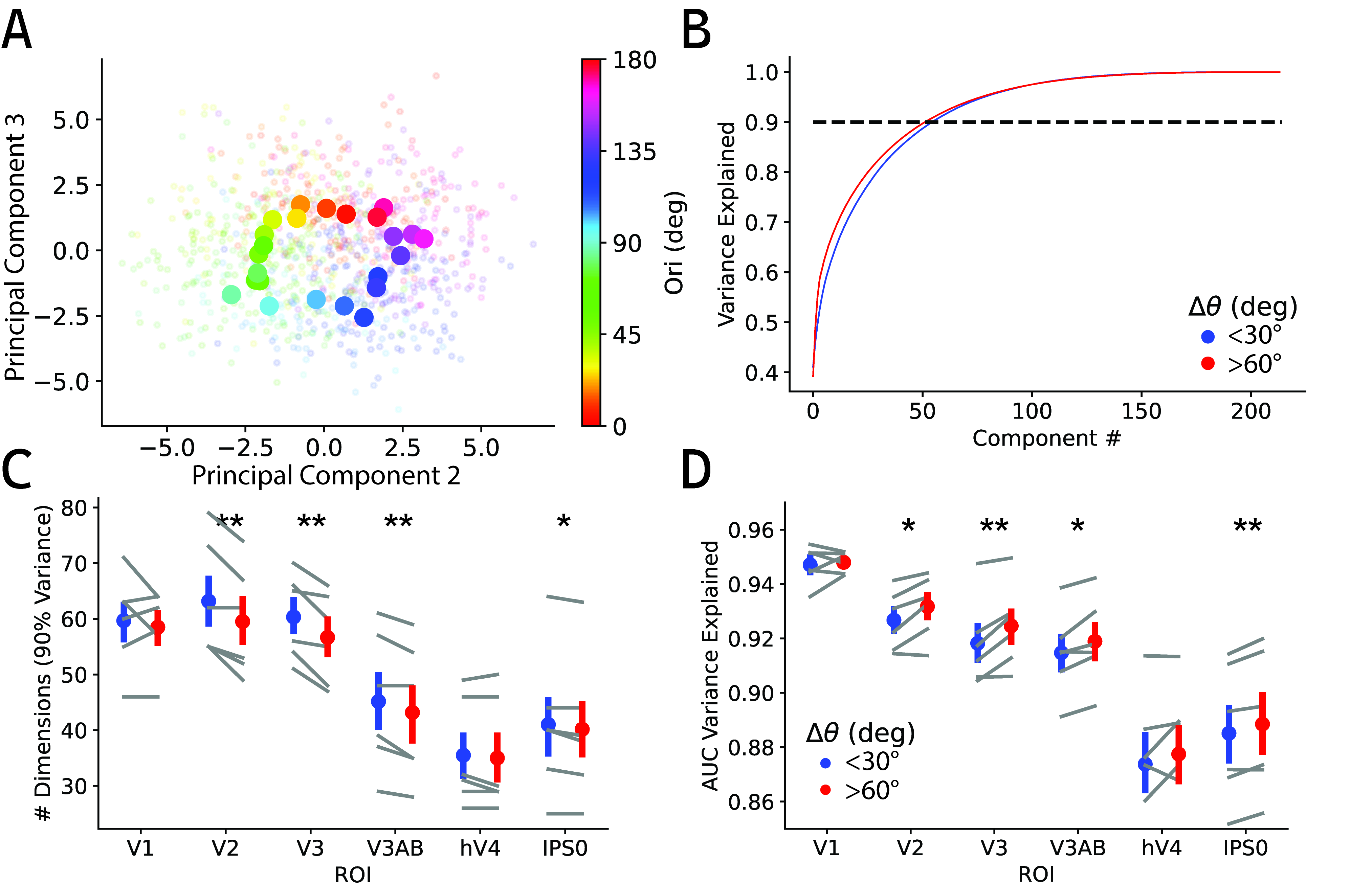

Supplement: S5 Fig — (A) We found that early principal components were correlated with the presented orientation, here presenting both individual trials as well as the average location for different orientation bins (large solid circles) for an example participant and ROI. (B) We performed PCA separately for trials following “close” and “far” trials, being careful to subsample the number of trials in the larger group. We then sorted the eigenvalues and examined the proportion of variance explained as a function of the number of components included separately for each group. (C) We found that it took significantly more components to explain 90% of the variance on the population activity following close versus far stimuli. This suggests that the representations in most visual areas occupy a higher dimensional space following close stimuli, but curiously not V1. Note that the total number of dimensions is shaped by the number of voxels included, so differences between participants/ROIs should not be interpreted with how these data were processed. (D) We additionally looked at the area under the variance curve to avoid any arbitrary effects of choosing 90% and found a similar effect (higher AUC implies lower dimensionality). Data and code supporting this figure found here: https://osf.io/e5xw8/?view_only=e7c1da85aa684cc8830aec8d74afdcb4. AUC, area under the curve; PCA, principal component analysis; ROI, region of interest. (TIF) [file pbio.3001711.s005.tif]

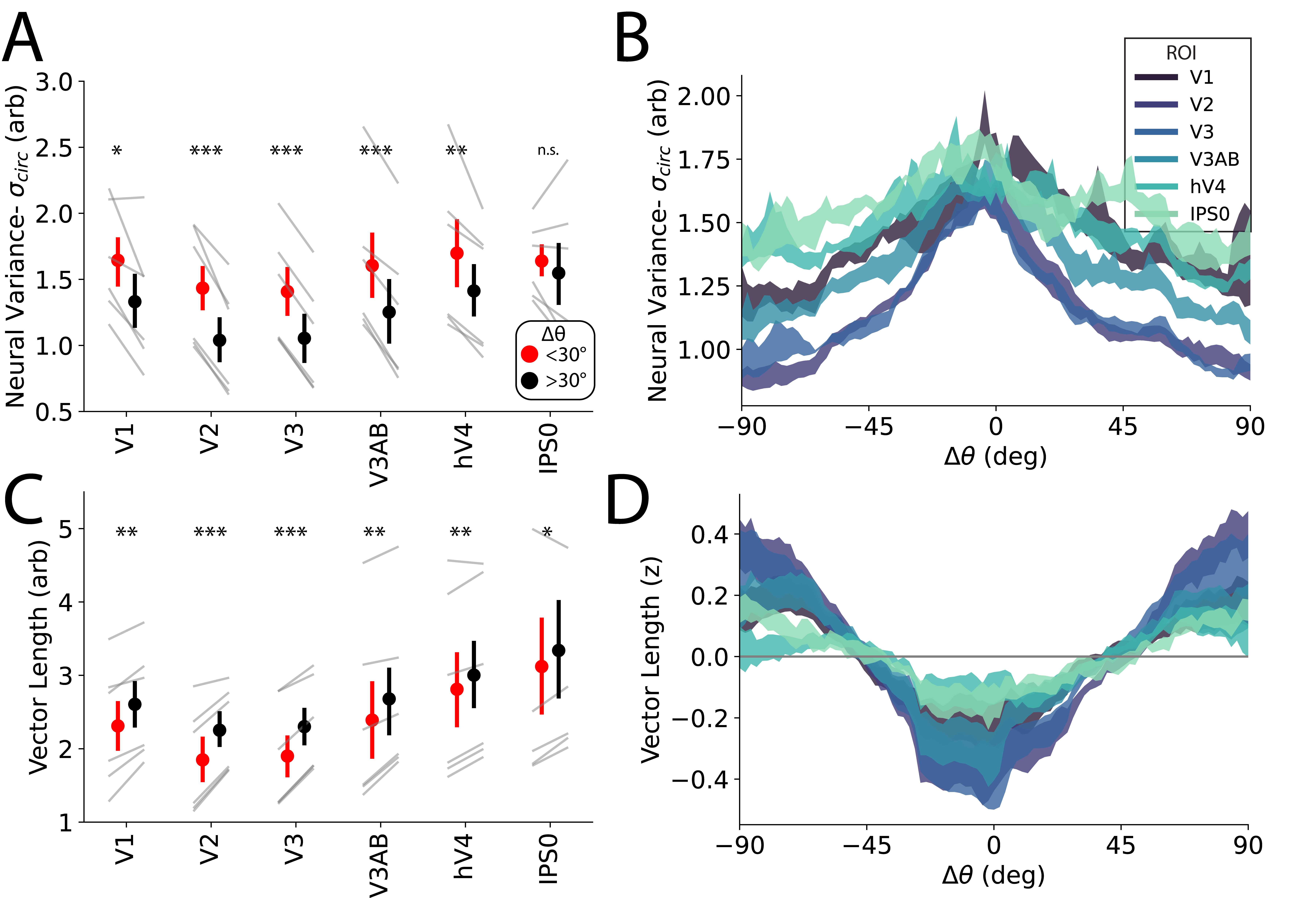

Supplement: S6 Fig — (A) σcirc of decoding errors is significantly greater for close (<30°) versus far (>30°) stimuli across early visual ROIs (see Neural variance). Points and error bars are mean ± SEM across participants; gray lines depict individual participants. Error bars depict SEM across participants. (B) Sliding σcirc for V1-V3 shows a monotonic relationship (± SEM across participants). (C, D) Same as A and B but measuring uncertainty directly measured from the single trial posterior (see Eq 8). Results are qualitatively very similar for both techniques. *, p < 0.05, **, p < 0.01, ***, p < 0.001. Data and code supporting this figure found here: https://osf.io/e5xw8/?view_only=e7c1da85aa684cc8830aec8d74afdcb4. ROI, region of interest. (TIF) [file pbio.3001711.s006.tif]

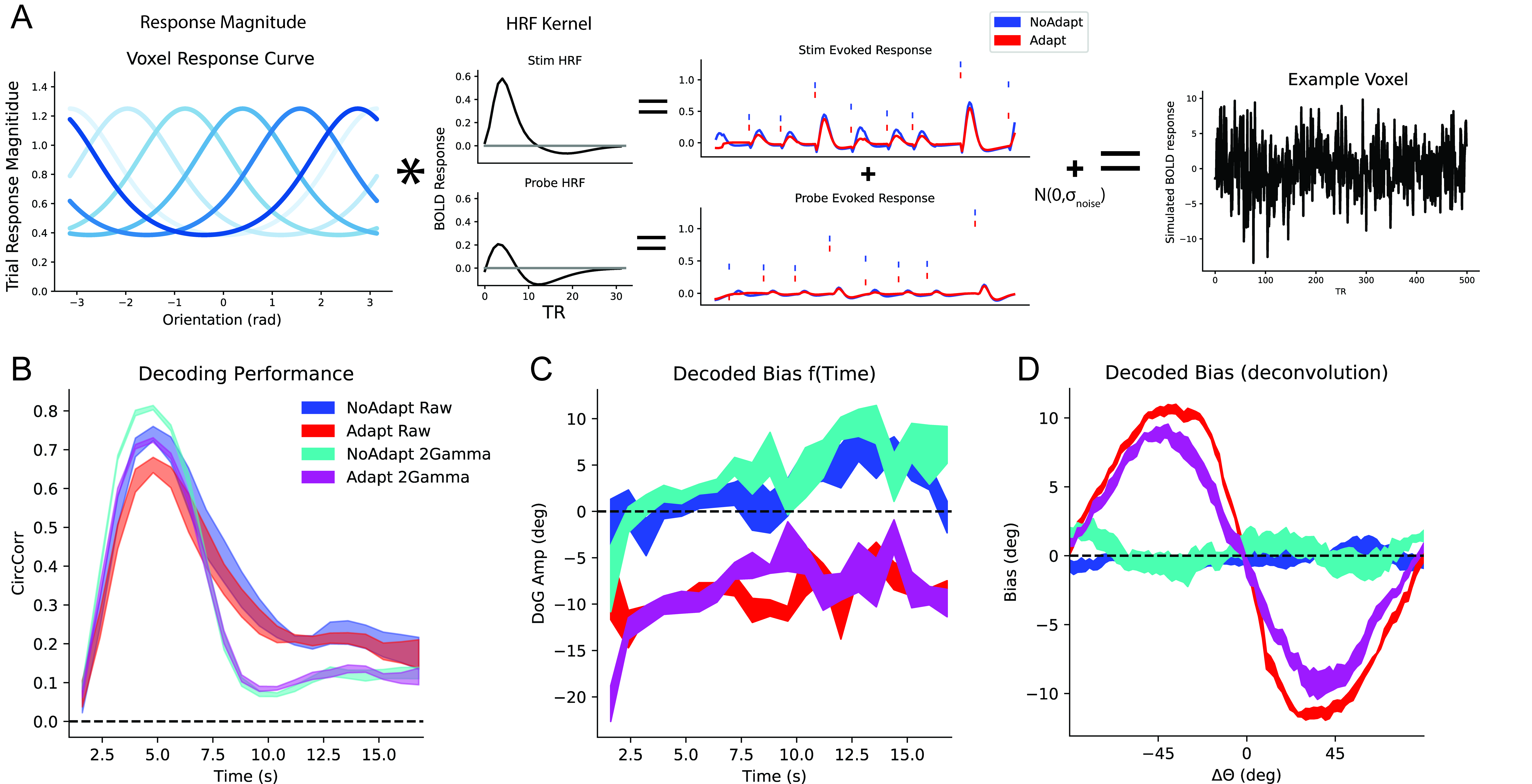

Supplement: S7 Fig — We first created a population of 32 voxels with uniformly distributed von Mises tuning curves. Note that for the purposes of this simulation, we are effectively treating voxels as neurons instead of a summation of the metabolic demands of many neurons. This shortcut comes from experience simulating voxel activity and finding decoding results are unaffected by such a shortcut while making results a bit simpler to understand (and faster to generate). The responses of each voxel were estimated by first generating a design vector based on the stimulus presentation times of both the stimulus and probe for a given participant with the amplitude of the response based on the defined tuning curves. This vector was then convolved with an empirically estimated HRF (both the raw output and when parameterized with a double gamma function) randomly sampled from voxels of the same participant to get the estimated evoked response to both the stimulus and the probe. These 2 signals were then combined along with gaussian noise to simulate the voxel response (A). Importantly, the tuning properties of these simulated voxels were unaffected by past stimuli so any biases found by applying our decoding techniques could reflect artifacts of our task design or analysis procedure. We additionally simulated BOLD responses with true adaptation in the underlying neural tuning. For simplicity, we simply attenuated the response to the current trial by 40% of the response to the previous trial while keeping all other stages of our analysis the same. We first applied a decoder across time to the epoched data and found a similar pattern to our empirical data with decoding performance following a parabolic shape before leveling off at some intermediate level, here utilizing HRFs from V3 voxels (B). This was true whether we used parameterized or raw HRFs and whether the simulation included adaptation. We next examined biases in our decoder as a function of stimulus history. With adaptation (red curves [file pbio.3001711.s007.tif]

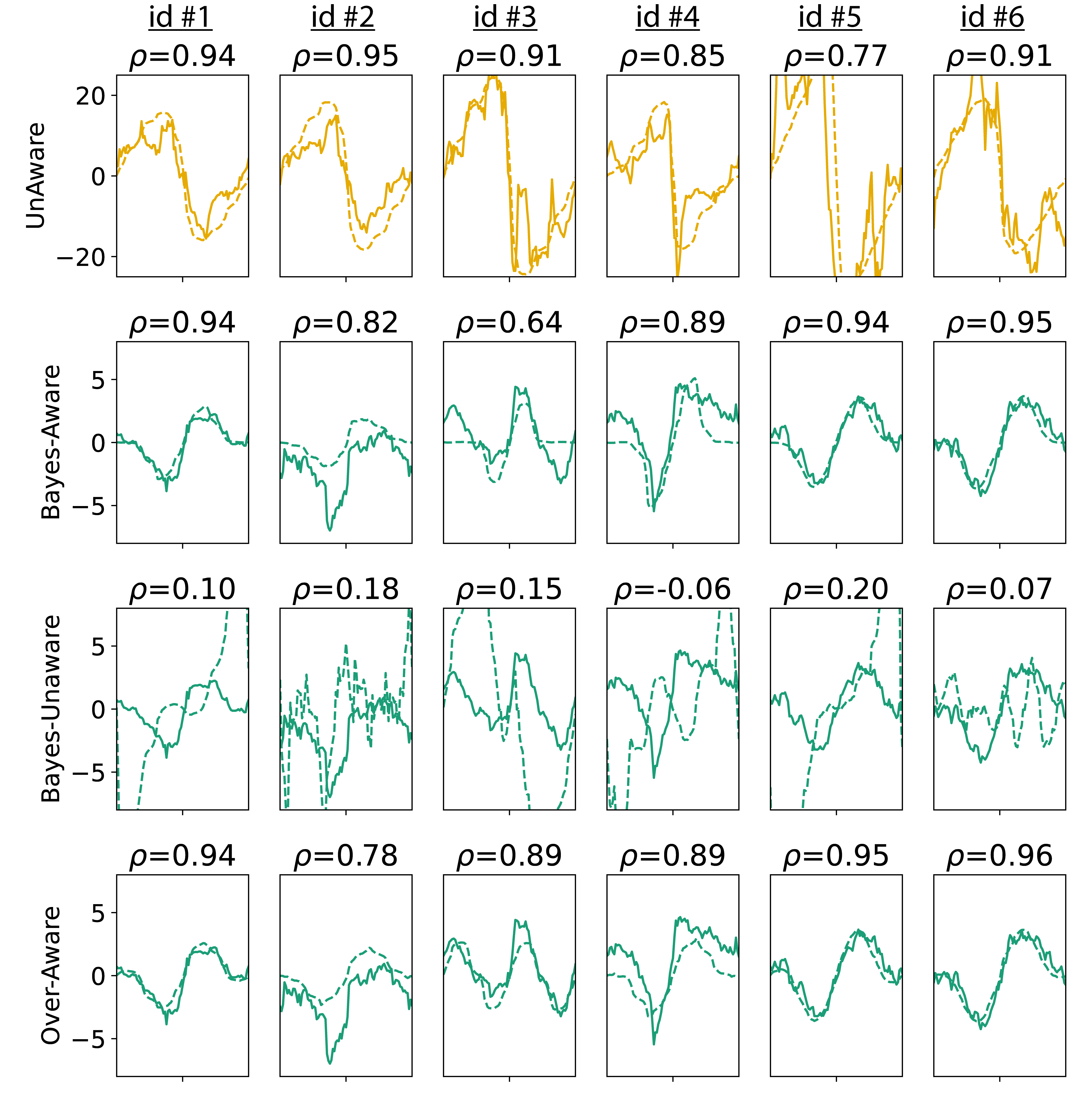

Supplement: S8 Fig — Solid lines correspond to empirical neural (yellow) or behavioral (green) bias; dashed lines correspond to model fits to BOLD decoding bias (Unaware model, A) or behavior (B–D). Model fits plotted are average of noiseless biases generated by models fit to each CV fold. Note that models are fit to raw data, not binned data presented here. Pearson correlations are reported above each fit between binned and model estimated bias. Data and code supporting this figure found here: https://osf.io/e5xw8/?view_only=e7c1da85aa684cc8830aec8d74afdcb4. (TIF) [file pbio.3001711.s008.tif]

**S1 Table**


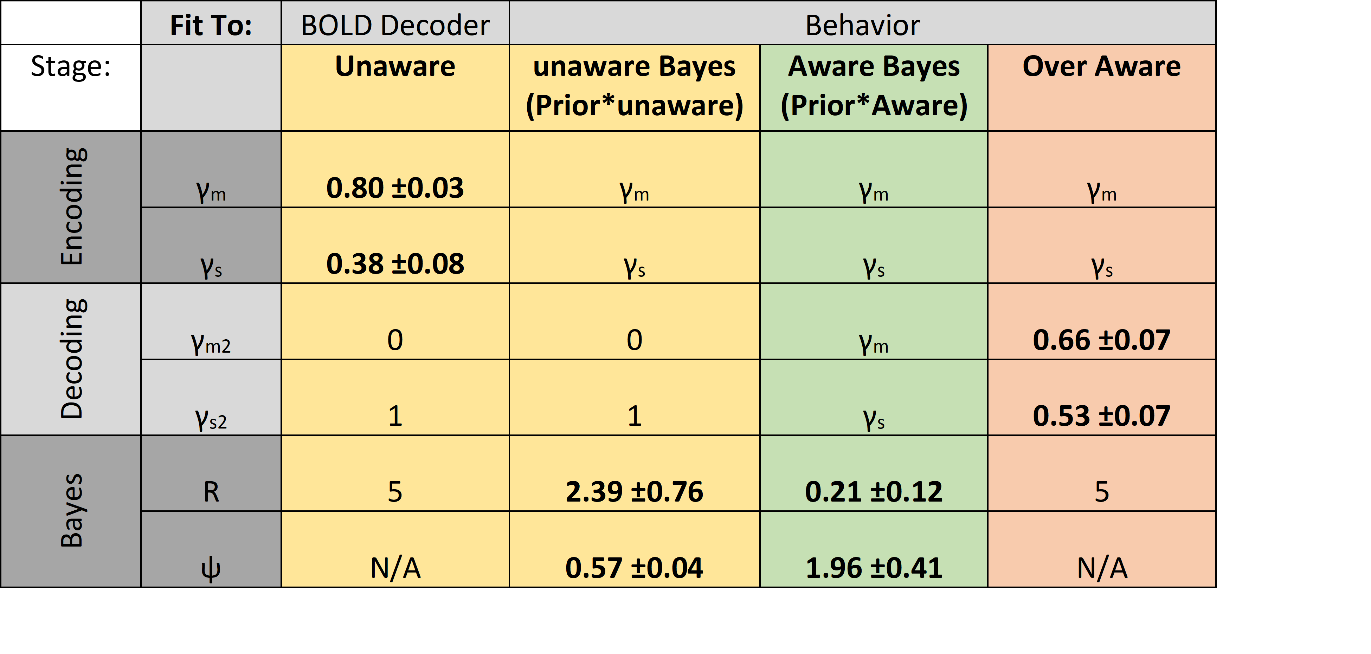

Supplement: S1 Table — Items with bold values indicate free parameters adjusted to fit empirical data (± SEM across participants). γm controls the amplitude, and γs controls the width of gain adaptation (Fig 4A). These parameters were fit by minimizing the RSS between the unaware decoder and the BOLD decoder output. γm2 and γs2 are the assumed adaptation parameters at decoding. These terms were either set to assume no adaptation (unaware), match the true amount of adaptation (aware) or are free parameters adjusted to maximize the likelihood of responses (overaware, Fig 4B). Last, R adjusts the average Poisson firing rate and ψ controls the variance of the prior distribution (Fig 4C). These parameters are adjusted for decoders using a Bayesian prior while R is set to the arbitrary value of 5 for non-Bayesian decoders (it has no effect on bias for non-Bayesian decoders). Increasing R increases the precision of the likelihood function and reduces the relative influence of the prior. Increasing ψ increases the range of Δθ over which the prior has an influence. Data and code supporting this figure found here: https://osf.io/e5xw8/?view_only=e7c1da85aa684cc8830aec8d74afdcb4. RSS, residual sum of squared errors. (DOCX) [file pbio.3001711.s009.docx]
