# Supplementary material for: Attractive serial dependence overcomes repulsive neuronal adaptation
Source: PLoS Biol. 2022 Sep 6;20(9):e3001711. doi: 10.1371/journal.pbio.3001711 (PMC9447932; doi:10.1371/journal.pbio.3001711)
Supplement: Attachment — Submitted filename: ReviewResponse.pdf [file pbio.3001711.s010.pdf]

UC San Diego

March 29, 2022,

General note on calcium data (old Figure 6).

All three reviewers expressed concerns relating to our re-analysis of publicly available the calcium data, especially in light of the large artifact showing attraction for the N+1 trial (i.e. attraction to an event that hadn't yet happened). We also found the artifact concerning and wanted to get a more concrete understanding of why it arises. The investigators that curated the dataset were quite helpful/responsive, but ultimately we only have access to the 'deconvolved' time series of estimated spikes and we are unable to access unprocessed, raw, data. Given the nature of the exponential filter that is used during the deconvolution, we were curious if this processing step introduced temporal-smearing artifacts that could account for the N+1 attraction that we observed.

We simulated calcium data by first generating orientation tuned activity in a population of 256 model neurons with circular normal tuning curves (matching our aware/unaware model parameters) with Poisson firing rates. Importantly we matched the trial timing of the task (750ms stimulus presentation, 250ms ISI), and our model had no adaptation or serial dependence built in. We then convolved this spiking activity with the assumed exponential decay kernel used in the deconvolution process. Lastly we applied the non-negative deconvolution package used in the original study (Suite2p: https://suite2p.readthedocs.io/en/latest/, specifically module dcnv.oasis) using the same settings as the original study. When we analyze this simulated data as a function of stimulus history, we observe an attractive bias to the future (N+1) stimulus that is an artifact of the processing pipeline. Critically, this pipeline also leads to an artifactual repulsion from the N-1 stimulus despite our simulated neural population not exhibiting any real adaptation. Because of this, we no longer include our re-analysis of the calcium imaging data as it is challenging to separate any neural effects from artifacts introduced during preprocessing.

Some additional notes. There are no artifacts introduced in the raw simulated fluorescence data, the biases only emerge after the non-negative deconvolution is applied. Additionally, the artifacts from the processed data go away with a longer ISI meaning this approach to analyzing calcium data is not wholly invalid, it just is in the context of answering questions about stimulus-history effects with 'sensitive' (slow decaying) calcium indicators and short ISIs.

Given the results of our simulated calcium data, we also ran simulations to determine if any choices in our fMRI processing pipeline could induce history-effects independent of true neural modulations. The sources of potential contamination are fundamentally different in the fMRI pipeline, so we can't compare the results directly (i.e., we don't use non-negative deconvolution, the temporal structure of the stimulus sequence is different, etc). Our simulation of BOLD data demonstrate that neither time course decoding or deconvolution based approach can result in the repulsive biases we observed in our BOLD data. These biases can only emerge in the presence of true neural adaptation. We now include the results of this simulation and believe these results further bolster our main conclusions.

Neuroscience Graduate Program and Department of Psychology
University of California San Diego • 9500 Gilman Drive #0109 • La Jolla, California 92093
858-246-1340 • tsheehan@ucsd.edu

UC San Diego

REVIEWS:

Reviewer #1: In this paper, Sheehan and Serences explore very convincingly the neural correlates of serial dependence in the visual cortex using fMRI. What is more, they use Bayesian modeling to back the proposal that while there is repulsion at the sensory level, downstream readouts can compensate for this repulsion, leading to the typical attraction. Finally, they (somewhat) replicate their findings in the mouse visual cortex.

I think this paper is of great interest to the cognitive neuroscience community, in particular those interested in serial dependence. I support the publication of this work at Plos Biology and I have some recommendations that I believe could improve the impact of the paper.

Overall, I think the paper is rather long, e.g. with control analyses given a full figure. My feeling is that this dilutes the major findings of the paper. I would go through it and reconsider moving each figure/panel to supplementary material (currently empty). Some obvious examples are Fig 3, 9. My personal experience was that I was rather tired towards the end of the paper, which is a pity since the model is a crucial component of the paper. The modeling par itself could be compressed in just one figure with clearer messages.

Thank you for this suggestion, we agree with this point on many levels. We have moved Figures 3, 4, 9 and Table 1 to the supplemental materials. We have additionally moved much of the description of the modeling work to the supplemental materials as well. We hope that this streamlines the paper and makes the main points more salient (and that we didn't remove too much information).

A more important concern is the choice of folding the serial dependence curves (as proposed in Barbosa and Compte 2020, not Barbosa et al. 2020; line 98) and *then* unfolding it again. The choice of folding serial dependence curves to improve power and remove systematic biases indeed assumes the curves are symmetric, but copy-pasting it back seems to be going too far. Relatedly, I would add to the supplementary material the unfolded curves.

In the spirit of your first comment to streamline the manuscript, we now just show the unfolded curves (rather than adding the full curves in addition to the folded curves). There are no qualitative differences in the results with the exception that, as expected, there is an increase in measurement variance. As the folded curves were for visualization purposes only and all statistics were performed on the unfolded data, nothing else in the text has changed. Note that we do still fold trial error when computing variance differences between close and far $\Delta\theta$ to avoid conflating opposing biases with larger variance and we highlight this choice in the related caption of Figure 1.

I think it is also important to discuss Hajonides et al paper (https://doi.org/10.1101/2021.10.31.46 6639). While I acknowledge that the current study was uploaded to biorxiv before, and has several major advantages, that paper is closely related with the current study and should be discussed for this submission.

We now include a discussion of these findings in the context of our study in the discussion, (line 401). This is in reference to the repulsive biases they find:

Neuroscience Graduate Program and Department of Psychology
University of California San Diego • 9500 Gilman Drive #0109 • La Jolla, California 92093
858-246-1340 • tsheehan@ucsd.edu

UC San Diego

"However, our observation of repulsive biases starting in V1 and persisting across later visual areas suggests that bottom-up adaptation may be a viable alternative explanation (which the authors also acknowledged). **Further support for this account comes from a recent MEG study showing that representations were repelled from past stimuli both within the current trial and from the previous trial (Hajonides et al., 2021). As in our study, this neural repulsion contrasts with attractive behavioral biases to the previous stimulus, suggesting sensory representations do not directly shape behavior even in simple sensory paradigms (Siegle et al., 2021)."**

In addition, we also reference their general ability to decode evidence for the previous stimulus (432): "The identity of the previous stimulus for spatial position and angle has been shown to be decodable from the spiking activity of single units in the frontal eye field (FEF) and large-scale activity patterns in human EEG **and MEG** (Papadimitriou et al., 2016; Fornaciai and Park, 2018; Bae and Luck, 2019; Bae, 2021; **Hajonides et al., 2021**)."

Below, I provide some more specific comments or suggestions that I recover as I go through the paper once again, thus not ordered by importance. I want to state clearly that these are just suggestions or ideas that popped into my mind as I read the paper and should not be taken as a case for acceptance/rejection.

Line 64. "Further, existing data from early visual " - which data? Please cite.

Thank you for catching this, we were referring to St. John-Saaltink et al., 2016. In reworking the introduction in line with reviewer 2s suggestions this sentence has been removed. A similar claim is now made in paragraph 4 of the introduction however (49):

"This perceptual account could arise from activity changes in early visual cortex, consistent with a fMRI study which measured early sensory biases that match 'attractive' behavioral reports (St. John-Saaltink et al., 2016)."

84-86. What correlates more with serial dependence, accuracy (as shown in the paper) or \delta \theta?

Response precision showed the strongest correlation with serial dependence, but we now expand this analysis to include these other factors which are also correlated (126).

"...This relationship was not an dependent on our response parameterization as we report found similar relationships between DoG amplitude and both accuracy (r=-0.41, Pearson correlation, p<.005) and average task difficulty $\delta\theta$ (r=.44, p<.005)."

123-. At this point I wondered what would be lost if only subjects with independent stimuli were considered for this study. If the results are upheld as it is claimed, I would remove completely the subjects with dependent stimuli. This would simplify the reading and length of the paper. If not, I would add a supplementary figure showing the full figure(s) only with/without those.

Neuroscience Graduate Program and Department of Psychology
University of California San Diego • 9500 Gilman Drive #0109 • La Jolla, California 92093
858-246-1340 • tsheehan@ucsd.edu

UC San Diego

While we agree this would simplify our presentation, we would also like the full dataset as collected. In lieu of completely removing these trials, we now present supplementary figures 2 and 3 excluding these trials for both the behavioral and BOLD cohort. In the interest of brevity, we additionally moved the statistics for this analysis to the supplement.

Behavior (130):
"A subset of participants completed a version of the experiment with inhomogeneities in their stimulus sequences (such that consecutive orientations were more likely to be between ±22.5 and 67.5° from the previous stimulus). **We repeated all of the above analyses excluding these participants and found all of our findings were qualitatively unchanged (Figure S2)."**

fMRI (147):
"A subset of these participants completed some sessions where consecutive stimuli were not strictly independent as they were more likely to be between ±22.5 and 67.5° from the previous stimulus (see Methods, Behavioral Discrimination Task, **4 out of 6 subjects had between 357-408 trials that were non independent accounting for between 40-50% of their trials and 32% of all trials completed). However, we replicated all of our main analysis excluding these sessions and found that our conclusion remained unchanged with the exception that our finding of reduced variance trended in the same direction but no longer reached significance (Figure S3)."**

As noted, and observable in figure S3A-B the variance pattern is no longer true for the fMRI cohort when removing these trials. We attribute this to a general loss in SNR by having fewer trials, emphasizing that reliably estimating variance requires more data than estimating the mean.

138. This is a weird finding, as many other studies have shown dependency on delay (Bliss et al 2017, Stein et al. 2020). I think this deserves a bit more unpacking / speculation about why you think you did not replicate this classical finding

Thank you for highlighting this important discrepancy, we now include a line on possible explanations (drawing on a suggestion by R2 to be careful about directly comparing orientation and other features like space, 365):

"It is curious that unlike some spatial working memory studies (Papadimitriou et al., 2015; Bliss et al., 2017; Stein et al., 2020), we did not find that behavioral biases increased with delay time. One possible explanation is that this phenomenon is actually unique to spatial working memory due to either a more consistent increase in sensory uncertainty of spatial location due to eye movements or a separate mechanism of memory maintenance that becomes more susceptible to proactive interference relative to orientation memories."

Figure 2. Why not include the fixation period in the decoding analyses? This would be particularly interesting when decoding previous-trial stimuli as in Baes, Barbosa, Papadimitriou. I suggest moving panel E to supplementary and adding the time course of previous trial decoding, in particular during the ITI.

Neuroscience Graduate Program and Department of Psychology
University of California San Diego • 9500 Gilman Drive #0109 • La Jolla, California 92093
858-246-1340 • tsheehan@ucsd.edu

UC San Diego

As we do not have a fixed ITI we have to time lock separately to both the current and previous trial. Expanding our current trial decoding traces, we additionally now show decoding of the N-1 stimulus time-locked to the current trial (Figure S3). For the task decoder, performance drops to chance when the next stimulus appears before recovering to above chance decoding. Notably, decoding performance for the decoder trained on a separate sensory stimulus task stays around chance suggesting stimulus representations during the subsequent trial are represented in a distinct code compared to sensory representations. We prefer to keep figure 2E in the main text as some readers have expressed particular interest in how our task decoder relates to sensory based coding.

148-150. Is this a one sided t test? It should be. Actually, given that you found a significant finding outside the scanner, the threshold should not be 0.05 (even if one sided). Consider using other analyses such as the Bayes factor.

We agree that the built in replication provides strong evidence and that we may not need to be as strict with our p-values. This type of behavioral replication is especially important in the case of some analysis which did not hold for all subsets of the data (for instance the change in response variance for the fMRI cohort excluding non-independent stimulus sequences as noted earlier). In general we prefer to use a more conservative approach when presenting statistics as it makes each finding easier to interpret on its own. We can also include Bayes factors if the reviewer feels strongly about this - we opted to use frequentist statistics here primarily because it is more familiar to people and because none of our conclusions hinge on a null result.

173. This is confusing and it seems very important for the interpretation of the fmri results. I would clarify this better.

We agree and thank you - the original text read:

“Unless otherwise specified, in future analyses we used the average of four TRs (spanning 4.8-8.0s) following stimulus presentation to avoid the influence of the probe stimulus (which came up ≥6s into the trial and thus should not influence responses in the 4.8-8.0s window after accounting for hemodynamic delay).”

We clarified when these timepoints were used and our rationale (172):

“...For all analyses not shown across time, we used the average of four TRs (spanning 4.8-8.0s) following stimulus presentation to minimize the influence of the probe stimulus (which came up ≥6s into the trial and thus should have a negligible influence on activity in the 4.8-8.0s window after accounting for hemodynamic delay, see Figure 5A).”

182. \mu_circ was not introduced.

We now clarify that \mu_circ refers to the circular mean of the decoding errors and point to the relevant methods section (187).

“...When measuring the bias **(circular mean, μ_circ, see Neural Bias)** of these decoding errors as a function of stimulus history (Δθ), we observed a strong repulsive bias reflecting neural adaptation (V3, Figure 2G yellow).”

Neuroscience Graduate Program and Department of Psychology
University of California San Diego • 9500 Gilman Drive #0109 • La Jolla, California 92093
858-246-1340 • tsheehan@ucsd.edu

UC San Diego

183. These neural serial dependence curves are beautiful. Curiosity: how would the serial dependence curves look if fully built from neural predictions? If I understand correctly, only the y axis is from neural data.

This figure (2G) depicts overlaid: neural bias (yellow) and neural variance (gray) as a function of change in stimulus orientation in the task. We could change the x-axis to reflect change in decoded orientation between trials, but such a depiction would be challenging to interpret and a somewhat circular presentation. However, if we are misunderstanding your point we would be happy to revisit this if you can explain further.

Figure 5 (and elsewhere) I would use + for future and - for previous.

Great point, thank you. We have remade these figures to reflect the more standard left-right chronology.

Figure 6. I would consider adding a schematic illustrating the task design. Why is C so different from B?

Thank you for pointing this out. We think that much of the confusion surrounding our procedure was due in part due to arbitrary decisions of how we presented the raw response probabilities. We emphasize that our parameterization is not particularly unusual and simply consists of fitting a mixture model (gaussian PDF + uniform guess rate) using a maximum likelihood procedure. In panel B we plotted "% response CW" but owing to how we designed our experiment, a greater proportion of CW responses would correspond (in general) to a CCW bias in the perception of the stimulus (because everything is relative to the probe that we presented at the end of each trial). As this display choice was arbitrary we now opt to present "% response CCW" in panel B so the transition to panel C is less confusing. We have additionally added a schematic, Figure S1, that better explains how we parameterized the responses.

Somewhat relatedly, we now clarify the coordinate space used in our study.

METHODS (569)
"We report degrees in a compass-based coordinate system such that 0° is vertical and orientation values increase moving CW (eg. 30° would point towards 1 o-clock)."

264. It is surprising the p value is 0.049. Judging by the figures the effect is so strong…

We no longer include the analysis of the calcium data as we think that analysis artifacts introduced by the nonnegative deconvolution could induce biases even in the absence of true neural effects. We describe this in more detail in a note at the beginning of our response to the reviews.

267. Why use a deconvolution kernel if it introduces spurious serial dependence with future trials? The origin of these future trials needs to be clarified, IMO. For example repeating the analyses without the deconvolution. And/or showing that it does not appear for purely behavioral plots as in Fig 5F.

We agree 100% but unfortunately we do not have access to the raw data for this study. See our note at the beginning of our reply letter for more details.

Neuroscience Graduate Program and Department of Psychology
University of California San Diego • 9500 Gilman Drive #0109 • La Jolla, California 92093
858-246-1340 • tsheehan@ucsd.edu

UC San Diego

Side note: In the current format, it seems the paper could finish right here. It is really challenging to embark on a completely different route (the Bayesian model). While I think this modeling work is crucial, I would consider moving most of it to supplementary and keeping only the main results/message. Note that in line 441 you summarize what is learned from the model. Going back to the figure, I feel it is possible to compress them substantially.

We have substantially reduced the section describing the modeling results by combining results into a single figure (now Figure 5) and moving the table and figure presenting individual fits to the supplemental section. Where possible we also reduced the length of the text by removing analysis or explanations that felt tangential to the main story.

291. m is undefined at this point.

We now define m in line (268):

"The unaware decoder assumes no adaptation has taken place and results in stimulus likelihoods p(m|θ) that are repelled from the previous stimulus (Figure 4C, yellow**, where m is the population activity at the encoding stage**)."

299. Not clear what a 'formal prior based on temporal contiguity' is

Thank you for catching this. This is building off of prior work that suggests the world is generally smoothly changing and continuous in those changes, and thus our perception is biased towards small changes (277).

"**We additionally built off of previous work showing stimuli are generally stable across time** by implementing a prior of temporal contiguity (van Bergen and Jehee, 2019). In our implementation, a Bayesian prior centered on the previous stimulus (Figure 4C, black) is multiplied by the decoded likelihood to get a Bayesian posterior (Figure 4C, bottom)."

Figure 8. Percept is undefined. More importantly, my intuition is that there is some circularity in the reasoning done in panel B. I would explicitly explain why there isn't.

Thanks for pointing out this typo. Panel A (now figure 5) caption now correctly indicates that the black outline reflects the empirically measured neural bias from V3. We have additionally clarified captions on new panels B and D.

We have removed the bar graph (old figure 8B and C) but still reference the analysis in the text. This analysis (quoted below) is confirming that our model of adaptation (which leads to a repulsive bias) is able to better explain the neural data than a model without adaptation. As our model is primarily concerned with connecting our neural data to behavioral findings, we did not particularly concern ourselves with comparing different forms of adaptation (eg. gain changes vs. shifts in tuning preference), in part because our voxel based recordings would be poorly suited to distinguish these models. We additionally added some context on how to interpret this analysis (quoted below, 293).

Neuroscience Graduate Program and Department of Psychology
University of California San Diego • 9500 Gilman Drive #0109 • La Jolla, California 92093
858-246-1340 • tsheehan@ucsd.edu

"The unaware readout provided a better fit to the outputs of our neural decoder than the **null alternative** of the presented orientation (t(5)=3.41, p=.01) because it captured a significant proportion of the variance in decoding errors as a function of Δθ (t(5)=7.5, p=.0007). **This analysis demonstrates that our adaptation model does a reasonable job of recovering our empirical decoding data (both of which use a decoder unaware of sensory history).**"

427. This is a very interesting point. I wonder if this could be quantified in the data (perhaps in future projects). It occurs to me to study dimensionality (e.g. with PCA) for different task conditions or subjects. Can one find a relationship between serial dependence and the dimensionality of neural representations?

Thanks for this suggestion. We now include a supplementary analysis where we find that the dimensionality of the space is indeed increased following close relative to far stimuli (Figure S5).

Results (193):
"In addition to the model-based analysis of responses in visual cortex, we also performed a model-free assessment of the dimensionality of activation patterns conditioned on the prior stimulus. Consistent with our main analysis, responses following close stimuli have a higher dimensionality than responses following far stimuli. This suggests that changes due to neural adaptation should assist pattern separation regardless of stimulus identity (see Dimensionality Analysis, Figure S5)."

Discussion (424):
"Thus, the biases introduced by adaptation may be beneficial in part because they expand the dimensionality of the representational space as we found in our recordings (Figure S5)."

432-433. I would cite these two papers separately. Barbosa et al 2020 is another possible citation here.

We have expanded our discussion to include additional related work including Barbosa et al., 2020 (426).

"We did not explicitly define how awareness of adaptation is implemented, **but it is clear that both attention to and conscious awareness of the previous stimulus are necessary for serial dependence to occur (Fischer and Whitney, 2014; Kim et al., 2020).** This is consistent with our model, and it suggests that some representation of information about stimulus history should be a minimum requirement for an aware decoding scheme. The identity of the previous stimulus for spatial position and angle has been shown to be decodable from the spiking activity of single units in the frontal eye field (FEF) and large-scale activity patterns in human EEG **and MEG** (Papadimitriou et al., 2016; **Fornaciai and Park, 2018**; Bae and Luck, 2019; **Bae, 2021; Hajonides et al., 2021**). We additionally demonstrate that information about the previous trial is encoded in patterns of fMRI activity in human visual cortex (Figure 2F), **but not in a sensory-like code (Figure S4AB).** These signals could potentially be represented concurrently with representations of the current stimulus in the same populations of sensory neurons **but in orthogonal codes analogous to what has been found for sequentially encoded items in primate prefrontal cortex and human EEG (Wan et al., 2020; Xie et al., 2022)**. An alternate account holds that representations of stimulus history are maintained outside of early visual areas, consistent with findings from mouse parietal and **primate prefrontal cortex** (Akrami et al., 2018; **Barbosa et al., 2020**). This anatomical segregation could disambiguate incoming sensory drive from

Neuroscience Graduate Program and Department of Psychology
University of California San Diego • 9500 Gilman Drive #0109 • La Jolla, California 92093
858-246-1340 • tsheehan@ucsd.edu

representations of stimulus history. **Critically, optogenetically suppressing non-sensory representations of stimulus history eliminated history effects, thus providing strong support for some form of an aware readout mechanism (Akrami et al., 2018).”**

480. This is a very interesting point. I would unpack it more in the results and abstract.

We now expand on this idea in the results section (326).

“More generally, this model has notable advantages that can lead to enhanced discrimination, reduced energy usage, and improved discrimination in naturalistic conditions over a static labeled-line representation.”

Finally, while reading both Hajonides and the current paper, it occurred to me that the repulsive neural correlates could be related to repulsive serial dependence seen when there is no current-trial delay (Bliss et al 2017). In general, I think it is good practice to have a delay-0 control condition when studying working memory. My prediction is that the neural repulsion seen here would correlate with the delay-0 repulsion. Unfortunately, this condition was not present in the fmri experiments and *I am not* suggesting new fmri data should be collected. However, please consider the following. Is there a correlation between attractive serial dependence for the protocols used inside and outside the scanner? If that is the case, one possibility to falsify my hypothesis would be to collect new, outside the scanner data, but including a delay 0 condition. Does that repulsion correlate with the neural repulsion? Again, this is pure speculation based on my own curiosity and I would not support a rejection case based on this 'missing' data.

This is a very interesting proposal. It is possible that the activity we are accessing with fMRI primarily corresponds to the ‘forward-sweep’ of activity and that the attractive biases we observe (as implemented in our model) arise either through feedback or in later brain regions. We are actively exploring how varying stimulus duration and delay shape attractive and repulsive biases, and especially looking at the role of backwards masking to minimize the influence of feedback on these representations. We are unfortunately unable to collect delay 0 conditions from our fMRI cohort as they have mostly moved away/ are no longer in our subject pool. That said, I think the general proposal that the observed neural biases could represent immediate perception of items may fit with our current understanding of the system and is an area we are actively pursuing.

Reviewer #2
A set of behavioral and fMRI experiments are presented on the neural basis of orientation serial dependence. This manuscript has many strengths: it uses sophisticated methods and presents data across species, even including reanalysis of mouse two-photon imaging data. It's an excellent manuscript in many ways, and it's very impressive too. My review is long, but that is because the manuscript is simultaneously excellent and yet the writing needs substantial revision. The paper is currently framed with the central question, essentially, "is serial dependence in visual cortex or outside visual cortex." This is a strawman. It would be better to reframe the paper to ask the unbiased but more important and open-ended general question: "Where can we find a manifestation of serial dependence in orientation". The results support a model in which there is a manifestation of orientation serial dependence in the read-out of information (wherever and however that happens).

Neuroscience Graduate Program and Department of Psychology
University of California San Diego • 9500 Gilman Drive #0109 • La Jolla, California 92093
858-246-1340 • tsheehan@ucsd.edu

UC San Diego

That is an interesting and novel idea, and it should be shared with the community. However, the discussion and conclusions about perception/decision/memory are not justified and the results are overinterpreted. That's easily fixed, though, because the manuscript doesn't test memory versus perception and doesn't need to be couched in the black-and-white debate about perception versus memory (just rephrase the question, as suggested above). The particular task and design (delay, working memory necessity, high contrast stimulus, full field presentation, etc, etc) favors finding an involvement of memory, favors producing adaptation, and could wash out any serially dependent pattern of activity in visual cortex, even if it were present. The null result—that visual cortex isn't involved--is hard to prove. As Kiyonaga et al, (TiCS, 2017) suggested, and as we now know from many subsequent papers, there are many forms of serial dependence, including in perception (appearance) and decision and memory (and in other domains like action too). There is also stimulus specific serial dependence (numerosity, face expression, attractiveness, timing, even audition and olfaction, etc etc). The experiments in this manuscript are interesting and worth reporting, but they need to be motivated and discussed in a more balanced way. With the revisions suggested below, I expect this will be a highly valuable paper that is certainly of broad interest to readers of PLoS Biology.

First, we thank the reviewer for the supportive and constructive comments. We have revised the framing of the paper extensively and think that it is much stronger as a result.

1. Abstract. Rephrase the sentence "Serial dependence is not implemented in visual cortex but rather by readout mechanisms that account for adaptation during encoding" to read "serial dependence is implemented in the readout mechanisms from visual cortex that account for adaptation during encoding". It is too strong (and loaded) to say that serial dependence is not "in" visual cortex (whatever that might mean). Who knows where the readout mechanisms are located, whether there is feedback, and whether the techniques here are able to isolate that readout is far from clear given that the mechanisms of readout itself are not fully specified or understood.

We agree that our phrasing was too strong and general. We now clarify that we are referring to the findings of our model which is meant to emphasize a novel account on how serial dependence *may* be implemented and not the only way it can occur in practice.

“We reconciled these opposing neural and behavioral biases using a model where both sensory encoding and readout are shaped by stimulus history. **First,** neural adaptation reduces redundancy at encoding and leads to the repulsive biases that we observed in visual cortex**. Second, our modeling work suggest that serial dependence is induced by readout mechanisms that account for adaptation in visual cortex. According to this account,** the visual system can simultaneously improve efficiency via adaptation while still optimizing behavior based on the temporal structure of natural stimuli.

2. The introduction introduces the terms "encoding" and 'readout': "We use the term "encoding" to refer to the initial conversion of external sensory information into neural activity patterns and the term 'readout' to refer to the readout of these encoded signals to shape behavior." These definitions are problematic and confusing. The "initial conversion of external sensory information" sounds more like transduction at the retina. Surely, there are "patterns" of responses in the retina and there is plenty of "neural activity patterns" in LGN and other places before V1 (not to mention feedback). These definitions just do not make sense, even loosely or operationally.

Neuroscience Graduate Program and Department of Psychology
University of California San Diego • 9500 Gilman Drive #0109 • La Jolla, California 92093
858-246-1340 • tsheehan@ucsd.edu

UC San Diego

We agree that our definition of encoding was unclear. We now try to reference specific patterns in the data throughout the text instead of over-using terms like "encoding". Thus, we removed that sentence, and the accompanying section of the intro now reads (line 20):

"For example, regularities can yield attenuated neural responses to frequently occurring stimuli **in early visual cortex** (adaptation), reducing metabolic cost and redundancy in neural codes (Dragoi et al., 2001, 2002; Benucci et al., 2013; Patterson et al., 2014; Fritsche et al., 2022). At readout, regularities might support the formation of Bayesian priors that can be used to bias **decision-making** in favor of higher probability stimuli (Stocker and Simoncelli, 2006; Cicchini et al., 2014; Wei and Stocker, 2015)."

3. Trying to define the "initial" or first "conversion" is not possible in this experiment and with these data. Trying to limit "encoding" to any particular level is also impossible. Encoding and read-out/decoding could happen at many levels and in many interacting ways. It seems that part of the problem is with the linear, hierarchical, and bottom-up way of thinking about visual processing, and (most problematic) the desire to simplify a straw man version of serial dependence as being either "in V1" or "not in V1". These are obviously oversimplifications.

As described above, we no longer attempt to define encoding using these overly specific terms. We continue to use the term readout but we try to be generally agnostic as to *where* such a biased readout would be implemented.

4. The abstract states that, "Previous behavioral studies suggest that serial dependence is implemented via modulations in visual cortex." In contrast to that quote, it is well-known in the literature that serial dependence requires attention (e.g., Fischer & Whitney, 2014; Kim, Burr, et al, 2020; Fritsche, 2019; Rafiei, et al., 2021; etc) and it occurs over a spatially broad region—far larger than any V1 RF (e.g., Fischer & Whitney, 2014; Fritsche, et al., 2017; Collins, Sci Rep, 2019; Cicchini et al., 2017; Manassi, Kristjansson, et al, 2019; etc). So, the idea that feedback (e.g., Cicchini et al., Curr Bio, 2021) and that more than V1 must be involved is not actually new (the only paper to really suggest V1, per se, was St. John-Saaltink et al). In fact, even Fischer's 2014 paper never mentions V1 or visual cortex, and the labeled line models they do mention could be implemented in the "read out" in a manner very similar to what is proposed in the current manuscript. To suggest--or imply--that the current results overturn a lot of psychophysical work is simply not justified. This should be acknowledged. Rather than framing the paper as a black-and-white "V1" versus "not-V1" the paper could incorporate a discussion of the richer and more nuanced prior psychophysical work.

Thank you for this suggestion. We now take a more holistic approach in our abstract and introduction sections in acknowledging that the serial dependence we observe (and the accompanying pattern of neural activity) may be specific to memory based biases and that the origins could be very different given different task parameters. Specifically, in the abstract we replace this sentence with one that reads:

"While behavioral studies have pointed to both perceptual and post-perceptual origins of this phenomenon, neural data that could elucidate where these biases emerge is limited."

Neuroscience Graduate Program and Department of Psychology
University of California San Diego • 9500 Gilman Drive #0109 • La Jolla, California 92093
858-246-1340 • tsheehan@ucsd.edu

Paragraphs 4 and 5 in the introduction now provide a more complete discussion of serial dependence literature (43, 56).

"In contrast to the repulsive **perceptual** biases typically associated with neural adaptation, **perceptual reports** are sometimes attracted to recently presented items – a phenomenon termed "serial dependence**". Studies utilizing low contrast oriented stimuli** suggest serial dependence can be perceptual in nature as it operates before a peripheral tilt illusion, impacts the perception of simultaneously presented items, biases perceptual reports even when no probe is presented, and does not require a working memory delay (Fischer and Whitney, 2014; Cicchini et al., 2017, 2021; Manassi et al., 2018; Murai and Whitney, 2021). This perceptual account could arise from activity changes in early visual cortex, consistent with a fMRI study which measured early sensory biases that match 'attractive' behavioral reports (St. John-Saaltink et al., 2016). This neural finding, however, is challenging to interpret as consecutive trials were always the same or orthogonal orientations which, by definition, cannot distinguish attractive from repulsive biases. **Related studies decoding past stimuli from EEG activity do not measure how current stimulus representations are biased, precluding a connection to behavioral biases (Fornaciai and Park, 2018; Bae and Luck, 2019; Bae, 2021).**

"Counter to studies reporting a **perceptual** locus of serial dependence which **utilized brief or low contrast stimuli**, other behavioral studies **utilizing high contrast spatial stimuli** have found that serial dependence does not emerge immediately but instead emerges only, and increases with, a working memory maintenance period (Papadimitriou et al., 2015; Bliss et al., 2017; Stein et al., 2020). This observation suggests that serial dependence could be implemented by a later readout or memory maintenance circuit (Papadimitriou et al., 2015; Bliss and D'Esposito, 2017; Pascucci et al., 2019; Barbosa et al., 2020). There is evidence that such a readout mechanism is Bayesian, as the influence of the "prior" (the previous stimulus) is larger when sensory representations are less precise due to either external or internal noise (Cicchini et al., 2018; van Bergen and Jehee, 2019). Thus, the existing behavioral evidence **suggests that serial dependence can operate both on perceptual and working memory representations** (Papadimitriou et al., 2015; Cicchini et al., 2017; Kiyonaga et al., 2017). **It is open question how and where past trial information interacts with incoming sensory and memory representations.**"

4. The authors use a delayed discrimination task. This design and approach are fine. However, other approaches also work to generate serial dependence and do not depend on delay or working memory. For example, there is serial dependence even in detection tasks (e.g., Murai & Whitney, Curr Bio, 2021) and simultaneous comparisons (e.g., Cicchini et al, JoV, 2017), among many other techniques (ratings, magnitude estimation, method of single stimuli, adjustment, etc etc). The delayed working memory dependent discrimination task is therefore not necessary to generate serial dependence, but it does favor finding something that hinges on that delay, and this risks biasing the results toward a memory-dependent form of serial dependence. That is perfectly fine—there is serial dependence that hinges on memory (e.g., Bliss et al., 2018)—but this does not rule out other forms of serial dependence. And, we do not know what would happen in visual cortex if another design were used.

This is a great point. At this stage it is not clear whether it is even useful to be grouping perceptual/immediate effects as belonging to the same phenomenon as memory dependent biases as is the norm in the literature.

Neuroscience Graduate Program and Department of Psychology
University of California San Diego • 9500 Gilman Drive #0109 • La Jolla, California 92093
858-246-1340 • tsheehan@ucsd.edu

We now acknowledge that our particular neural findings are possibly very specific to our memory dependent paradigm. We now acknowledge that our findings are specific to the data generated by our design and will not necessarily translate to studies using low contrast stimuli.

Discussion (338)

"The critical new insight revealed by the model is that only readout schemes that account for adaptation can explain the attractive behavioral bias **observed in our paradigm**. More generally, our BOLD data argue against an **early sensory origin of serial dependence for orientation and instead suggest that serial dependence is driven by post-perceptual or mnemonic circuits (Pascucci et al., 2019; Barbosa et al., 2020). However, because we used a paradigm that required working memory, our results may not generalize to other situations in which serial dependence is observed even in the absence of a memory delay (Fischer and Whitney, 2014; Cicchini et al., 2017; Collins, 2020; Murai and Whitney, 2021).** Thus, future work is needed to better understand the role of sensory representations **in paradigms with low contrast stimuli, that do not require a memory delay period, and that utilize other features besides orientation.**"

Discussion (371…390)

"Evidence for an **early sensory** origin of serial dependence comes from an fMRI study with low contrast stimuli and a short (500 ms) delay period which reported that both behavioral responses and V1 representations were more precise following a matching stimulus (St. John-Saaltink et al., 2016). This departure from our own finding could be driven by the stimuli which were rendered to have a very high uncertainty… Further work examining neural biases using **low contrast stimuli** will shed further light on a **potential role of coding changes in sensory cortex driving serial dependence**."

5. How do we know that serial dependence "manifested in V1" was not simply washed out by a stronger negative aftereffect? Several papers have demonstrated that the same stimuli can generate both positive and negative aftereffects (depending on things like duration, contrast, size, noise, etc), and that they can be additive. This even goes back to Fischer 2014, as the authors acknowledge, who found negative aftereffects for either long adapting durations or ignored stimuli. Many other papers have found additive positive and negative aftereffects as well. How can we rule out the possibility of superimposed aftereffects that are just dominated by one or the other in this particular experiment?

This is an interesting possibility. Behavioral studies can push attractive biases to repulsive by increasing stimulus contrast/duration (Fischer and Whitney, 2014, Manassi et al., 2018) and have demonstrated temporally distant stimuli have distinct impacts on perception relative to immediate stimuli (Fritsche et al., 2020). As for neural effects, an interesting study by Patterson et al., 2013 recording primarily from layer 2/3 in V1 did find distinct effects of brief (0.4s) stimulus presentations relative to long (4s, 40s). Notably for large stimuli, brief stimuli led to the repulsion of tuning curves (population code attraction) while long stimuli led to the attraction of tuning curves (population repulsion, Figure 3B). This is an area we are actively exploring in current work and we now better address these points in the discussion on page 22.

Discussion (373)

"**This departure from our own finding could be driven by the stimuli that were rendered to have a very high uncertainty. Past work studying adaptation in non-human primates found repulsive patterns following long**

Neuroscience Graduate Program and Department of Psychology
University of California San Diego • 9500 Gilman Drive #0109 • La Jolla, California 92093
858-246-1340 • tsheehan@ucsd.edu

(4s and 40s) but attractive patterns following short (0.4s) stimulus presentations suggesting stimulus duration may have a large influence on how past stimuli shape future sensory processing (Patterson et al., 2013)."

6. The method of estimating an effective PSE is novel but somewhat dense and confusing. Although the authors took some pains to try to explain how "response bias" relates to "behavioral bias," the relationship is still obscure. What is estimated response error as a function of orientation (not difference in orientation)? On first blush it appears that the response bias is a repulsion, but that's not clear and is easily misinterpreted. How do Fig1B-C relate to each other? Is panel B the best way to convey the steps in the analysis to get to panel C?

Thank you for pointing this out. We think that much of the confusion surrounding our procedure was due in part due to arbitrary decisions of how we presented the raw response probabilities. We emphasize that our parameterization is not particularly unusual and simply consists of fitting a mixture model (gaussian PDF + uniform guess rate) using a maximum likelihood procedure. In panel B we plotted "% response CW" but owing to how we designed our experiment, a greater proportion of CW responses would correspond (in general) to a CCW bias in the perception of the stimulus (because everything is relative to the probe that we presented at the end of each trial). As this display choice was arbitrary we now opt to present "% response CCW" in panel B so the transition to panel C is less confusing. We have additionally added a schematic, Figure S1, that better explains how we parameterized the responses.

Somewhat relatedly, we now clarify the coordinate space used in our study.

METHODS (569)

"… We use a compass coordinate system for reporting stimulus orientation such that 0° is vertical and orientation values increase moving CW (eg. 30° would point towards 1 o-clock)."

7. There is a concern about mirror flipping the data because the stimulus distribution is already circular and it's not clear why mirror flipping or merging data should be necessary unless there is some other systematic response bias lurking in the data (e.g., consistent biases reporting clockwise, response bias toward cardinal orientations, repulsion away from cardinal orientations, etc etc). Flipping and merging can cause something that is not serial dependence to look like serial dependence (e.g., central tendency biases). That a previous study did it is not very convincing justification (and some previous papers did this kind of thing for linear dimensions, mistaking a central tendency bias for serial dependence). Perhaps the raw non-mirrored data can be presented in supplemental material?

Rather then add additional materials, we now present all curves as unfolded (this was also suggested by R1). We removed the associated section from the methods section. We note that in the original submission we performed statistics on the unflipped curves so nothing else in the text changed.

8. How do we know the "read-out" does not happen in signals directly from V1? How do we know where the "read-out" happens?

Neuroscience Graduate Program and Department of Psychology
University of California San Diego • 9500 Gilman Drive #0109 • La Jolla, California 92093
858-246-1340 • tsheehan@ucsd.edu

UC San Diego

We certainly cannot say where the biased readout happens beyond indicating that we did not find attractive biases in any of the regions we examined. We think the strongest argument against the possibility of direct read-out from V1 is that repulsive patterns continue to be visible across the visual hierarchy (including areas V3 and IPS0). That said, we don't have good leverage on the question of where read-out happens as we cover in the discussion, past studies suggest representations of past trial information (which would be necessary for 'aware' readout) have been found in parietal as well as prefrontal cortex so these regions may be good candidates (Discussion 438):

"An alternate account holds that representations of stimulus history are maintained outside of early visual areas, consistent with findings from mouse parietal and **primate prefrontal cortex** (Akrami et al., 2018; Barbosa et al., 2020). This anatomical segregation could disambiguate incoming sensory drive from representations of stimulus history."

9. P. 4. The proposed distinction between serial dependence as "sensory" versus "working memory" is an oversimplified strawman. As Kiyonaga et al pointed out in what is now an old paper (2017, TiCS), there could be many forms of serial dependence (memory, perception, action, etc), and the notion of serial dependence as being black and white "either/or" is simply not supported in the literature. There are ample demonstrations of serial dependence without working memory (e.g., Cicchini et al., 2017; Murai & Whitney, 2021; Liberman et al., 2014), and also serial dependence that does depend on working memory (Papadimitriou et al, 2015; Bliss et al., 2017; etc). The mechanisms of adaptation and serial dependence need not be restricted to just one processing stage (e.g., see Collins, JEP, 2021; Liberman, et al., 2014, 2018; Taubert, Alais, & Burr, 2016; etc), and they may depend on stimuli and task. Serial dependence does not just happen for orientation, it happens for numerosity, face expression, identity, object shape, and other stimulus dimensions. I'm not aware of any paper that suggests that all of these serial dependencies are the same or that they all occur in V1.

We agree and have substantially re-worked our intro/discussion sections to better emphasize that our study primarily applies to memory dependent serial dependence. For example, in the introduction (page 5, line 64) we now clarify:

"Thus, the existing behavioral evidence **suggests that serial dependence can operate both on perceptual and working memory representations** (Papadimitriou et al., 2015; Cicchini et al., 2017; Kiyonaga et al., 2017). **It is open question how and where past trial information interacts with incoming sensory and memory representations.**"

10. Fig. 2F. Decoding the previous stimulus replicates prior work and is useful. Can you try the same analylsis on the N+1 stimulus (future)? It shouldn't work, so it's a good confirmation that other artifacts aren't lurking.

This is a great control analysis, which we now include (page 11, 181):

"As a control analysis, we attempted but were unable to decode the identity of the next stimulus using the same procedure (Figure S3F)."

Neuroscience Graduate Program and Department of Psychology
University of California San Diego • 9500 Gilman Drive #0109 • La Jolla, California 92093
858-246-1340 • tsheehan@ucsd.edu

11. Fig 2H. The direction of the effect is a bit confusing because it doesn't seem to match the caption description ("Decoded variance is significantly less.." and yet it is opposite panel 2B).

Thank you for catching this typo. We have fixed the caption. In line with your larger point for distinguishing 'perceptual' biases from general behavioral biases, we also changed the y-axis for panel A to imply the biases are 'behavioral' and not necessarily 'perceptual' as previously presented.

(Figure 2) "H: Decoded variance is significantly **greater** for $|\Delta\theta|<30°$"

12. Fig. 6D X axis labels confusing. Positive and negative numbers are confusing when using the words "trials back". What is the n-1 trial? Is that the positive 1 "trial back" or is that the -1 trial back (with a double negative)? Try using "back" and "forward" or some other way to disambiguate what is meant.

Figure 6 (now figure 3, panels E and F) has been revised to reflect normal chronology so the influence of different trial numbers increases left to right (this was also suggested by R1).

13. The large, noise-free, high contrast stimulus could bias the results toward finding a negative aftereffect in visual cortex (sensory adaptation). When contrast is increased and noise reduced, serial dependence tends to be weaker (e.g., Cicchini, et al; see also Kim & Alais, Vis Res, 2021), and if there are additive or separable effects in V1, the serial dependence might not show up. The flickering (phase reversal every 250 msec) could have a similar impact, improving measurement of adaptation and diminishing power to measure a neural signature of serial dependence. The long duration (compared to behavioral studies) of the stimulus (1 sec) could have a similar effect.

We agree that our stimulus parameters certainly increase the chance of us observing repulsive effects relative to other serial dependence studies that utilize briefer and noisier stimuli. We now touch upon the role of stimulus contrast and duration in the discussion (373).

"This departure from our own finding could be driven by the stimuli which were rendered to have a very high uncertainty. Past work studying adaptation in non-human primates found repulsive patterns following long (4s and 40s) but attractive patterns following short (0.4s) stimulus presentations (Patterson et al., 2013) suggesting stimulus duration may have a large influence on how past stimuli shape future sensory processing."

14. The point above casts doubts on the generality of statements like, "This repulsive pattern suggests that serial dependence is not a direct result of attractive biases in early sensory areas." Statements like that are not more valid than other conclusions based on the inconsistency of the measured visual cortex responses with perception. For example, the wildly strong adaptation (repulsion) effects (Fig 3C) in early visual areas (perhaps helped by high contrast, full field stimuli) also do not produce a negative perceptual (tilt) aftereffect, but we would hesitate to conclude from these data alone that "V1 has no direct influence on perception of orientation." Maybe that is true and maybe it isn't. What we can take away is that the neural patterns definitely do not match behavior.

Neuroscience Graduate Program and Department of Psychology
University of California San Diego • 9500 Gilman Drive #0109 • La Jolla, California 92093
858-246-1340 • tsheehan@ucsd.edu

We agree that this statement is too general given there are instances where serial dependence could occur under very different circumstances (with much weaker neural adaptation). We have removed this sentence from the results section.

15. Fig. 3. The variability in the "neural bias" between subjects is enormous and the source of it is unclear but is concerning. Some observers have around 7 deg peak bias and some observers have well over 15 deg, and one has well over 30 deg of bias. Despite that variability, the behavioral effect seems relatively consistent across participants.

(Now Figure S4). We agree that there is substantial variability in the neural bias curves. Some subjects have lower SNR and the resulting bias curves may be harder to interpret. That said, the overall pattern of repulsive biases is highly consistent and observed in all subjects, and we emphasize that presenting raw bias curves for individual participants is not typical and – to our knowledge – unseen in existing studies examining neural correlates of serial dependence. For example, other studies just report a single bias value collapsing across all stimulus changes and subjects (eg. St. John-Saaltink et al., 2016, Hajonides et al., 2021) whereas here each datapoint corresponds to a subset of trials from a specific ROI and participant. When using all datapoints to constrain our estimate as in fitting DoG curves this variability is reduced (see subject dots in Figure 2I, 3B). Thus, we don't have any basis for comparison yet in the literature and it will be interesting to see if future studies also observe such variability and if so whether it systematically maps onto meaningful changes in behavior.

16. p. 32. "For some behavioral participants (n=9) delay and ITI were varied between 0.5-7.5s without notable effects on performance." This result seems at odds with the papers cited on page 4. Any ideas why?

All of the studies that we are aware of that show strong delay time modulations are spatial in nature. We have run many experiments in the lab – in addition to those reported here – looking for such effects using orientation, and in our experience the role of delay seems to be more "all-or-none". Specifically, we do not observe serial dependence in experiments using very short delays (0.5-1s) but consistently observe attractive biases with delays over 3s. One possibility is that orientation representations do not decay much beyond the initial loss of iconic memory while spatial representations decay more continuously (perhaps due to eye movements, or the natural statistics of eye movements). We now discuss this possibility more (page 21, line 365):

"It is curious that unlike some spatial working memory studies (Papadimitriou et al., 2015; Bliss et al., 2017; Stein et al., 2020), we did not find that behavioral biases increased with delay time. One possible explanation is that this phenomenon is actually unique to spatial working memory due to either a more consistent increase in sensory uncertainty of spatial location due to eye movements or a separate mechanism of memory maintenance that becomes more susceptible to proactive interference relative to orientation memories."

17. Fig. 5. It is not clear what, if anything, we can take away from the very long duration repulsive effects. They have little or no tuning, they don't match behavior (null result) and the conclusion from that figure was that "Together this suggests that adaptive biases may be much stronger and more persistent than previously thought." Previous behavioral studies have reported long term negative and positive aftereffects (e.g., Gekas, et al., JoV, 2019). However, the results here do not speak to that.

Neuroscience Graduate Program and Department of Psychology
University of California San Diego • 9500 Gilman Drive #0109 • La Jolla, California 92093
858-246-1340 • tsheehan@ucsd.edu

UC San Diego

We agree that the long duration adaptation effects are surprising, and note that we become increasingly under-powered in our analysis as we look for effects further back in time (as our blocks are only 17 trials/ ~5 minutes). Our motivation for examining biases from stimuli further back in time was to provide further confidence that our observed repulsive biases were not an artifact of the HRF evoked on previous trials. We are primarily interested in addressing neural biases here and as we do not find any significant behavioral biases beyond the N-1 trial further discussion of related biases extending back many trials feels tangential. The sentences quoted have been removed in favor of a more relevant conclusion driven by our data (251).

"Together these analyses suggest that our observed biases are driven by adaptation in the underlying neural population and provide additional evidence that behavior is not directly linked to early visual representations."

18. "This suggests that the adaptive biases are more persistent than often thought and provide further support for a neural basis of our findings." Again, this is a stretch and a very tenuous connection. Fig 5 shows a repulsion that lasts 52+ sec (or more, since only 3-back was reported) and that is far longer than 10 sec in the cited paper. It's not comparable and whether an artifact causes the long duration suppression in the fMRI data remains unclear. Additionally, there are many previous behavioral studies on the time course of serial dependence, but the time course of the neural repulsion here doesn't agree with those.

The quoted sentence (along with that entire section) has been removed (see note at beginning of review). We still address these concerns however. Our motivation for citing the Fritsche 2021 (now published Fritsche 2022) paper stems from contextualizing our neural findings (which we agree are dissimilar from behavioral serial dependence findings). Our larger point is that repulsive biases in neural representations have been found in a study from just this year to extend much further than is conventionally expected. Fritsche 2022 had a much shorter ISI and these biases persist across as many as 20 intervening stimuli so the relevance here seems high. The final version of their manuscript includes a bias that extends back 22s and importantly, like our own biases, decays exponentially. We note that while the biases around 52s back are surprising, the magnitude follows a steady reduction from the N-1 stimulus and they occur with only two intervening stimuli of an otherwise fixated gray screen making them consistent with a standard exponential decay of stimulus history. We now address this in the text:

"These neural biases were surprisingly persistent and are in line with recent studies which have found adaptation signatures extending 22s in mouse visual cortex spiking activity (Fritsche et al., 2022). It is not clear why our effects persist even longer, but it is likely driven in part by the long ISIs, resulting in fewer intervening stimuli compared to the paradigm utilized in Fritsche et al., 2022.

We have also added a new supplemental modeling analysis to further address any concerns relating to artifacts driving our results (Supplemental Figure 7).

19. Fig 6. Apparently, the most significant effect (n+1 positive effect) is actually an artifact. That's a concern and the explanation for it is hand waving and reveals more about a problem with the experiment design and analysis. Although most of that wasn't under the authors' control, whether to present it and how to reanalyze and interpret it is.

Neuroscience Graduate Program and Department of Psychology
University of California San Diego • 9500 Gilman Drive #0109 • La Jolla, California 92093
858-246-1340 • tsheehan@ucsd.edu

UC San Diego

We found this odd as well and dug into it more. Please see our note at beginning of our response to the reviewers, and we have opted to exclude the calcium data from the manuscript.

20A. Fig. 9. The model gets close to making predictions about individual differences, which is an excellent goal. However, the actual predictions for the behavioral data (e.g., Fig 9B or 9D) are nearly identical across observers and rather untethered from the neural (yellow) data. That suggests different observers predict each other pretty well. Whether individual differences are captured in any way is unclear (granted that 6 subjects is too few for any individual differences, but there should be some observer specific correspondence; the change in variability between observers is unclear).

Our Bayes-aware models effectively undoes any bias introduced by adaptation so there no longer is a direct influence of neural activity on behavioral bias. However, the over-aware model does reveal some notable individual differences as we note in the text, particularly with subject id #3 where it able to achieve peripheral repulsion. In line with an earlier suggestion, we now present unfolded data curves and present data that we believe show clear individual differences in model fits (now Figure S8). Individual perceptual variance curves, owing to the larger amount of data needed to estimate the variance compared to the mean, are too noisy to be reliably compared.

20B. How is the "neural" data in Fig 9A related to the "neural" data in Fig 3C? Shouldn't they match or correspond closely? Some observers do appear to have similar or corresponding data (ID#5) and some don't (ID#4 looks way off), but it is unclear why.

Thank you for pointing this out. We originally used a wider convolution kernel in Figure 3C. We now use the same kernel in all figures (32° width) and present the “unfolded” curves.

20C. Fig 9. The model works here, but the time course in the neural data is far off what has been reported in the literature (and far off that found in the behavioral data here) so whether the model would generalize is unclear.

Our model is not implemented in a time dependent manner in part because our ISIs were a minimum of 11s in our experiment. As we now emphasize in our discussion, analogous adaptation findings have now been reported in single unit recordings and it is unclear how these findings would change if stimulus conditions were matched across studies (e.g. by having longer ISIs in single unit study). Our model does not explicitly cover long range behavioral effects in part because we did not observe them (perhaps due to our long ISI). Our model could be adapted and expanded but for this study we are best suited to well describe effects extending just a single trial back in time.

20D. Whether the results would hold in other more "perceptual" tasks (working memory is not required for serial dependence) is also unclear but has not been tested. That limit should be acknowledged.

We agree that studying these distinct forms of serial dependence is a worthwhile future direction. As noted above, we now address this point in the discussion (373).

Neuroscience Graduate Program and Department of Psychology
University of California San Diego • 9500 Gilman Drive #0109 • La Jolla, California 92093
858-246-1340 • tsheehan@ucsd.edu

"This departure from our own finding could be driven by the stimuli that were rendered to have a very high uncertainty. Past work studying adaptation in non-human primates found repulsive patterns following long (4s and 40s) but attractive patterns following short (0.4s) stimulus presentations suggesting stimulus duration may have a large influence on how past stimuli shape future sensory processing (Patterson et al., 2013)… Further work examining neural biases using low contrast stimuli will shed further light on a potential role of coding changes in sensory cortex driving serial dependence."

21. Discussion section. "More generally, our BOLD data argue against an early sensory or 'perceptual' account of serial dependence and instead suggest that serial dependence is driven by post-perceptual or mnemonic circuits." This is not valid. The referenced "perceptual" account does not and need not make claims about V1 or other cortical areas being the seat of perception or the source of serial dependence. Those papers are psychophysical: the "perceptual" account is that "perception" is impacted by serial dependence. The current manuscript does not speak to that debate at all. The primary question in this manuscript was, as stated above, "Where can we find a manifestation of [orientation] serial dependence?" The authors do succeed in proposing a reasonable and interesting answer to that question—the read out of information might underlie at least one type of serial dependence—but, the authors have not tested anything about perception (e.g., appearance) versus decision
versus memory (independent of perception). The sentence should be deleted and the rest of the discussion rebalanced to reflect the much richer literature, including the references in this review (listed below).

We agree that our findings do not directly address whether serial dependence impacts the perceptual experience of the observers. We have substantially reworked our introduction and discussion sections to present a more balanced perspective and integrated findings from many of the cited studies in the text. We removed the reference to perceptual account in the highlighted sentence (339):

"More generally, our BOLD data argue against **an early sensory origin of** ~~**or 'perceptual' account of**~~ serial dependence **for orientation** and instead suggest that serial dependence is driven by post-perceptual or mnemonic circuits (Pascucci et al., 2019; Barbosa et al., 2020).

Of particular relevance, in the discussion we now also state (335):

"As our experiment always utilizes a working memory delay, it is unclear if the bias towards past stimuli is driven by a change in their perception of the stimulus itself or instead somehow biases their comparison with the probe stimulus only after the working memory maintenance period."

22. The perceptual account of serial dependence is a psychophysically based theory (or set of theories: Cicchini et al; Liberman et al; Collins; Alais et al; Fischer et al; Fornaciai et al; etc), it is not that V1 is responsible for serial dependence (of orientation, numerosity, faces, or anything else). The debate about whether serial dependence is in perception and/or decision and/or memory (see Kiyonaga et al, 2017) is not addressed by invoking V1 or ruling it out (or ruling out any other cortical area, for that matter). Whether serial dependence is "in" V1 or in the "read-out" from an area like V1 does not address or disprove the substantial behavioral literature.

Neuroscience Graduate Program and Department of Psychology
University of California San Diego • 9500 Gilman Drive #0109 • La Jolla, California 92093
858-246-1340 • tsheehan@ucsd.edu

UC San Diego

We agree and no longer present our findings as disambiguating the perceptual nature of serial dependence.

For example intro (64):
"Thus, the existing behavioral evidence suggests that serial dependence can operate both on perceptual and working memory representations (Papadimitriou et al., 2015; Cicchini et al., 2017; Kiyonaga et al., 2017)."

Discussion (392):
"In contrast to studies favoring ~~**a perceptual**~~ **an early sensory account** – and more in line with the paradigm and findings reported in this manuscript – a single unit recording study in non-human primates used high contrast stimuli and a longer working memory delay (1.4-5.6s) (Papadimitriou et al., 2016)."

23. General comment. Serial dependence has been reported in many dozens of papers and in many domains, from numerosity (Corbett et al; Cicchini et al; Fornaciai et al; etc), orientation, face expression (Liberman et al., 2018; Alais et al., 2021; etc), face identity (Kok et al., 2017; Turbett et al., 2019; Liberman, et al, 2014; etc), shape (Manassi et al., 2021), texture (Manassi et al., 2017), attractiveness (Taubert et al, 2016; Taubert & Alais, 2016; Van der Burg et al., 2019; Xia et al, 2016; etc), to timing, audition (Ho et al., 2019), cross modal perception, and now even smell (Van der Berg, et al., 2021). Several papers have reported dissociations between these dimensions (e.g., Fornaciai & Park, 2019; Collins, 2021; others), showing that serial dependence is not monolithic; there is not a single homunculus or module for serial dependence—it is domain/stimulus/task specific. This manuscript only tests orientation, and the results only apply for orientation. Throughout the manuscript, the text should be specific that the experiments, results, and conclusions are only about orientation serial dependence, and the result may not generalize to any other stimulus.

Throughout the manuscript, we now emphasize the specific domain that we studied (orientation). For example in the opening paragraph of the discussion we now write (339):

"More generally, our BOLD data argue against an early sensory origin of serial dependence **for orientation** and instead suggest that serial dependence is driven by post-perceptual or mnemonic circuits (Pascucci et al., 2019; Barbosa et al., 2020). **However, because we used a paradigm that required working memory, our results may not generalize to other situations in which serial dependence is observed even in the absence of a memory delay (Fischer and Whitney, 2014; Cicchini et al., 2017; Collins, 2020; Murai and Whitney, 2021). Thus, future work is needed to better understand the role of sensory representations in paradigms with low contrast stimuli, that do not require a memory delay period, and that utilize other features besides orientation."**

24. p. 26. "However, others have found that serial dependence is repulsive at very short delays and only becomes attractive when items are held for an extended time in working memory (Papadimitriou et al., 2015; Bliss et al., 2017)." These cited papers are about stimulus position, not orientation, and do not hold for the current experiment. First, the authors own data (delay, ITI dependence) doesn't seem to support this statement (and if it does, that needs to be reported). Second, other papers have not found these delay effects for orientation (or other stimuli). Third, if the authors want to rely on unrelated (non-orientation) stimuli for their preferred interpretation (selectively citing Bliss and Papadimitriou, for example), then they need to be

Neuroscience Graduate Program and Department of Psychology
University of California San Diego • 9500 Gilman Drive #0109 • La Jolla, California 92093
858-246-1340 • tsheehan@ucsd.edu

thorough and balanced about citing papers on serial dependence across many domains (shape, faces, numerosity, tone, timing, etc etc).

We now additionally reference studies that use stimuli more similar to our own and provide separate evidence for serial dependence not being perceptual in the context of high contrast stimuli (406).

“Behavioral studies using similar high contrast orientation stimuli to our own have also shown that responses are attracted to past decisions and repelled from past stimuli, further suggesting these attractive biases do not emerge in early sensory areas (Pascucci et al., 2019; Sadil et al., 2021).”

We cite the spatial position papers to provide a clear example of serial dependence not directly impacting perception. We now (as mentioned previously) include a discussion of why we believe this particular finding of the attractive effect increasing with delay is not found in our study. Spatial position stimuli presented on an annulus have many critical similarities with orientation stimulus spaces making them much more relevant to our work than the cited examples. For example they both occupy a circular space that affects the relationship of feature similarity and is clearly distinct from monotonic features such as numerosity, tone, and timing. Spatial position is also easily parameterized and can be naturally decoded from primary visual cortex in a way that these other features cannot. Thus we do not think that spatial location tasks are ‘unrelated’ to orientation relative to these other features. Instead, we think that these studies of spatial position are relevant to discuss if we are more careful to articulate that our conclusions are based on orientation alone (and we now try to be more careful with this, as noted in replies to other comments).

25. p. 26. "Consistent with the perceptual account, an fMRI study with low contrast stimuli… consistent with a perceptual origin" The cited paper is not "consistent" or "inconsistent" with the perceptual account. It didn't psychophysically measure anything about perception and didn't tie the results to perception (it also suffered from the problems the authors describe at length). It suggested V1 as a possible correlate of orientation serial dependence, and the current results disagree with that. But, the current results do not speak to perception vs memory vs decision. It is not justified to equate V1 to "perception": the "perceptual account" of serial dependence is not the same thing as the "V1 account" of serial dependence. This confusion permeates the manuscript. The results in this manuscript might reject a V1 account (V1 as the sole source of serial dependence) but it does not test or reject the "perceptual account[s]".

We agree with these important distinctions and have reworked all references to the perceptual nature of serial dependence to fit in line with existing evidence and that found in our data. The cited sentence has been re-written as (371):

“Evidence for an early sensory origin of serial dependence comes from an fMRI study with low contrast stimuli and a short (500 ms) delay period which reported that both behavioral responses and V1 representations were more precise following a matching stimulus (St. John-Saaltink et al., 2016).”

26. p. 26. "Thus, while some behavioral studies show convincing evidence of serial dependence emerging immediately with perception, the existing neuronal evidence supporting this account is inconclusive." It is true that there is relatively little neurophysiological evidence for the origins of serial dependence in any stimulus domain (despite the fact that it is found in hundreds of behavioral papers on all manner of stimuli). There are

Neuroscience Graduate Program and Department of Psychology
University of California San Diego • 9500 Gilman Drive #0109 • La Jolla, California 92093
858-246-1340 • tsheehan@ucsd.edu

several uncited papers, however, that have investigated possible neural sources (e.g. using EEG; Fornaciai & Park, 2018, 2020; Bae, Cer Cor Comm, 2021; and MEG too, etc) and some of these papers do find early signatures of serial dependence. The real problem here is that being "immediate" is not required for "perception". It is not the immediacy of the effect that matters, it's the fact that perception, per se, is impacted. "Perception," for example, is operationalized in some previous studies as "appearance" (e.g., Collins, 2020; Cicchini,et al., 2017). If the authors want to redefine "perceptual" as something else, they need to justify that, but it's not valid to simply equate "perceptual" to "V1".

We have expanded our discussion to cover these papers and no longer present neural evidence as clarifying debates between perceptual and post-perceptual origins of serial dependence (382).

"…Related work has shown the ability to decode the previous stimulus from EEG activity patterns (Fornaciai and Park, 2018; Bae and Luck, 2019; Bae, 2021) but it is important to note that our study also showed robust decoding of the previous stimulus that did not also correspond with an attractive bias in the neural representation of the current stimulus (Figure 2F-G, S4). This is because the representations of current and past stimuli are not necessarily stored using the same code. Thus, while previous neural studies have argued that serial dependence emerges in visual cortex, no study has demonstrated an attraction towards the previous stimulus dependent on feature similarity consistent with behavioral biases. Further work examining neural biases using low contrast stimuli will shed further light on a potential role of coding changes in sensory cortex driving serial dependence."

All three studies report the ability to decode the identity of the previous stimulus. This is distinct from demonstrating an attractive bias in the representation of the representation of the current trial (as our own study demonstrates). Only systematic biases in the current trial's representation would constitute evidence for a neural marker of serial dependence.

27. "Thus, in line with our findings, behavioral and neuronal studies using high contrast stimuli have found results consistent with a post-perceptual account of serial dependence." Again, this misleadingly equates V1 (or some early cortical visual area) with "perception", does not operationally define what "post-perceptual" is, and it ignores a very large existing literature. The results in this manuscript are, in fact, important and interesting, but the conclusions and terms used need to be specific and more carefully chosen. Put another way, even if the fMRI results had been flipped and supported V1 as the sole source of serial dependence, that would not support a "perceptual account" because none of the experiments here provide a new or critical test of "perception" versus memory, decision, etc. The experiments identify a plausible model and neural mechanism in the readout of information from visual cortex. This could hold for serial dependence in perception and/or decision and/or memory. Best to remain agnostic about that.

Thank you for these points and we agree. We have modified that sentence accordingly (as well tried to be mindful of these points in the rest of the text, line 411).

"Thus, in line with our findings: behavioral, neuronal, and **modeling studies utilizing high contrast stimuli in working memory paradigms consistently point to attractive effects emerging in either memory or decision-making circuits and not early sensory areas**."

Neuroscience Graduate Program and Department of Psychology
University of California San Diego • 9500 Gilman Drive #0109 • La Jolla, California 92093
858-246-1340 • tsheehan@ucsd.edu

Review References

Alais, D., Xu, Y., Wardle, S. G., & Taubert, J. (2021). A shared mechanism for facial expression in human faces and face pareidolia. Proceedings of the Royal Society B, 288(1954), 20210966.
Bae, G. Y. (2021). Neural evidence for categorical biases in location and orientation representations in a working memory task. NeuroImage, 240, 118366.
Bliss DP, D'Esposito M (2017) Synaptic augmentation in a cortical circuit model reproduces serial dependence in visual working memory Bazhenov M, ed. PLOS ONE 12:e0188927.
Cicchini, G. M., Mikellidou, K., & Burr, D. (2017). Serial dependencies act directly on perception. Journal of vision, 17(14), 6-6.
Cicchini, G. M., Benedetto, A., & Burr, D. C. (2021). Perceptual history propagates down to early levels of sensory analysis. Current Biology, 31(6), 1245-1250.
Collins, T. (2019). The perceptual continuity field is retinotopic. Scientific reports, 9(1), 1-6.
Collins, T. (2020). Serial dependence alters perceived object appearance. Journal of Vision, 20(13), 9-9.
Collins, T. (2021). Serial dependence occurs at the level of both features and integrated object representations. Journal of Experimental Psychology: General.
Fornaciai, M., & Park, J. (2018). Serial dependence in numerosity perception. Journal of vision, 18(9), 15-15.
Fischer J, Whitney D (2014) Serial dependence in visual perception. Nat Neurosci 17:738-743
Fornaciai, M., & Park, J. (2019). Serial dependence generalizes across different stimulus formats, but not different sensory modalities. Vision research, 160, 108-115.
Fornaciai, M., & Park, J. (2020). Neural dynamics of serial dependence in numerosity perception. Journal of Cognitive Neuroscience, 32(1), 141-154.
Fornaciai, M., & Park, J. (2018). Attractive serial dependence in the absence of an explicit task. Psychological Science, 29(3), 437-446.
Fritsche, M., & de Lange, F. P. (2019). The role of feature-based attention in visual serial dependence. Journal of vision, 19(13), 21-21.
Fritsche M, Mostert P, de Lange FP (2017) Opposite Effects of Recent History on Perception and Decision. Curr Biol 27:590-595.
Gekas, N., McDermott, K. C., & Mamassian, P. (2019). Disambiguating serial effects of multiple timescales. Journal of vision, 19(6), 24-24.
Ho, H. T., Burr, D. C., Alais, D., & Morrone, M. C. (2019). Auditory perceptual history is propagated through alpha oscillations. Current Biology, 29(24), 4208-4217.
Kim, S., Burr, D., Cicchini, G. M., & Alais, D. (2020). Serial dependence in perception requires conscious awareness. Current Biology, 30(6), R257-R258.
Kim, S., & Alais, D. (2021). Individual differences in serial dependence manifest when sensory uncertainty is high. Vision Research, 188, 274-282.
Kiyonaga, A., Scimeca, J. M., Bliss, D. P., & Whitney, D. (2017). Serial dependence across perception, attention, and memory. Trends in Cognitive Sciences, 21(7), 493-497.
Liberman, A., Fischer, J., & Whitney, D. (2014). Serial dependence in the perception of faces. Current biology, 24(21), 2569-2574.
Manassi, M., Ghirardo, C., Canas-Bajo, T., Ren, Z., Prinzmetal, W., & Whitney, D. (2021). Serial dependence in the perceptual judgments of radiologists. Cognitive research: principles and implications, 6(1), 1-13.

Neuroscience Graduate Program and Department of Psychology
University of California San Diego • 9500 Gilman Drive #0109 • La Jolla, California 92093
858-246-1340 • tsheehan@ucsd.edu

Manassi, M., Kristjánsson, Á. & Whitney, D. Serial dependence in a simulated clinical visual search task. Sci Rep 9, 19937 (2019). https://doi.org/10.1038/s41598-019-56315-z
Manassi, M., Liberman, A., Chaney, W., & Whitney, D. (2017). The perceived stability of scenes: serial dependence in ensemble representations. Scientific reports, 7(1), 1-9.
Murai, Y., & Whitney, D. (2021). Serial dependence revealed in history-dependent perceptual templates. Current Biology.
Taubert, J., Van der Burg, E., & Alais, D. (2016). Love at second sight: Sequential dependence of facial attractiveness in an on-line dating paradigm. Scientific reports, 6(1), 1-5.
Taubert, J., & Alais, D. (2016). Serial dependence in face attractiveness judgements tolerates rotations around the yaw axis but not the roll axis. Visual Cognition, 24(2), 103-114.
Papadimitriou C, Ferdoash A, Snyder LH (2015) Ghosts in the machine: memory interference from the previous trial. J Neurophysiol 113:567-577.
Rafiei, M., Hansmann-Roth, S., et al. Optimizing perception: Attended and ignored stimuli create opposing perceptual biases. Atten Percept Psychophys 83, 1230-1239 (2021). https://doi.org/10.3758/s13414-020-02030-1
Taubert, J., Alais, D., & Burr, D. (2016). Different coding strategies for the perception of stable and changeable facial attributes. Scientific reports, 6(1), 1-7.
Turbett, K., Palermo, R., Bell, J., Burton, J., & Jeffery, L. (2019). Individual differences in serial dependence of facial identity are associated with face recognition abilities. Scientific reports, 9(1), 1-12.
Van der Burg, E., Rhodes, G., & Alais, D. (2019). Positive sequential dependency for face attractiveness perception. Journal of vision, 19(12), 6-6.
Van der Burg, E., Toet, A., Brouwer, A. M., & van Erp, J. B. (2021). Sequential Effects in Odor Perception. Chemosensory Perception, 1-7.
Xia, Y., Leib, A. Y., & Whitney, D. (2016). Serial dependence in the perception of attractiveness. Journal of vision, 16(15), 28-28.

Reviewer #3

This manuscript uses psychophysics, fMRI, two-photon imaging, and computational modeling to better understand the serial dependence effect in visual perception. Behaviorally, they find a healthy serial dependence effect, wherein the perceived orientation of a stimulus is 'drawn to' the previously presented stimulus orientation in a sequence. However, using both fMRI and analyses of two-photon imaging data in mice, they find evidence in early visual cortex that does not exhibit this attractive effect, instead observing instances in which the orientations are repulsive, a pattern more consistent with an adaptation-based account for visual encoding. The authors interpret these results as evidence against the hypothesis that the perceptual serial dependence effect is a direct result of computations within visual cortex, but rather the result of a readout mechanism that is potentially 'aware' of the adapted state of the visual system. The breadth of the methods used to address this phenomenon is impressive, to say the least, and the rigor is generally very high. I have a few questions and concerns that could improve the clarity of the manuscript, which are detailed below.

Neuroscience Graduate Program and Department of Psychology
University of California San Diego • 9500 Gilman Drive #0109 • La Jolla, California 92093
858-246-1340 • tsheehan@ucsd.edu

1. As the authors know, the sluggish nature of the hemodynamic response is problematic when making claims regarding recent past events influencing measures of present events, and how it was potentially tainting the measure of present events with preceding events —an effect that complicates measures of history-dependent serial orientation effects. Different orientations produce different, sometimes significant, changes in BOLD response, and as the authors note, this is a potential serious concern, and they do their best to assuage this concern. I find as a whole this is satisfactory, particularly taking into account the two-photon CA imaging results. However, there is one analysis that was not entirely clear, and I think the authors could better unpack. The authors deconvolve their data, and then fit these deconvolve responses with a double gamma, from which they then use the weights for subsequent analyses. But, one thing I need clarification on is whether this was a deconvolution that was stimulus orientation dependent, or across all orientations. I ask because I want to better understand whether they observed any qualitative changes between various orientation conditions at all. Some more clarification of this analysis would help me better understand how appropriately it addresses the issue.

We have extensively expanded our explanation of how we go about estimating HRFs and how we use these to estimate single trial response magnitudes (copied below). We do not fit separate HRFs for different orientations as this would preclude us from then using the magnitudes of our estimated evoked responses in our orientation decoder. Our method does assume the shape of the evoked response is consistent across orientations and that only the magnitude varies. While this assumption may not always hold true, the value in having an estimator of evoked responses across orientations that is unbiased should outweigh any fit improvements by allowing the HRF shape to vary.

From Methods, page 35, line 670:

Estimating average voxel HRFs using deconvolution

Because we are measuring the effects of previous stimuli on responses to the current stimulus, we did an additional analysis to quantify any influence of overlapping Hemodynamic Response Functions (HRF) that last for 20-30s (e.g. the "undershoot" that happens approximately 8-18s post-stimulus; see Figure 3A). To account for overlapping HRFs, we used deconvolution to estimate the average univariate response separately in each voxel in each ROI by modeling the responses to both the stimulus and probe for 30 TRs (24s) post-stimulus (Dale, 1999; Glover, 1999). We created a design matrix (rows x columns = total number of TRs x 30) with the first column containing ones corresponding to the onset TR of each stimulus (and zeros elsewhere). Subsequent columns were the same vector shifted forward in time by one TR. Following the same procedure, another design matrix was defined for the probe onset times. These matrices were stacked with a column of ones added for each run as a constant term, yielding a final design matrix X of dimensions (total number of TRs x (60+number of blocks)). We created a related matrix of voxel activity Y (total number of TRs x number of voxels) by concatenating responses in each voxel across blocks. We then estimated the HRF by performing least squares regression using the normal equation:

$$h = (X^T X)^{-1} (X^T Y). \quad [1]$$

The resulting weights corresponded to the average timecourse of the HRF evoked separately by the stimulus and the probe across all trials. We note that this HRF is estimated independent of the orientation of the

Neuroscience Graduate Program and Department of Psychology
University of California San Diego • 9500 Gilman Drive #0109 • La Jolla, California 92093
858-246-1340 • tsheehan@ucsd.edu

presented stimuli as we wanted to use these estimates to then decode orientation dependent changes in activation patterns. For each voxel we then parameterized the HRF using a 6-parameter double gamma function using scipy.optimize.minimize so that we could use the voxel-specific HRF model in a GLM to estimate the response magnitude in each voxel on each trial. We excluded the 11% of voxels which failed to converge on a solution.

Estimating trial-by-trial responses using parameterized voxel HRFs

For each voxel, we then created a design matrix Xv (rows x columns = total number of TRs x (number of trials * 2 +number of blocks)) with each column a delta function centered at the onset of the stimulus (or probe). We then regressed this matrix onto the (total # of TRs) vector Yv of voxel activity using equation 9. This resulted in a simultaneous estimation of the trial-by-trial magnitude of responses to each stimulus grating and each probe which was repeated for each voxel to allow voxel specific HRFs to be utilized in the creation of Xv. The resulting activity pattern associated with each stimulus was used in the same manner as the raw time course of the BOLD response to train and test an IEM, and the resulting estimates should be largely independent of linear contributions of previous stimuli (Dale, 1999)."

To further assuage concerns about any interactions between nearby trials, we separately added a modeling section where we simulate voxel responses using both raw and parameterized voxel HRFs using the actual trial sequences completed by participants (Figure S7). This analysis importantly revealed that repulsive biases can only emerge in both the raw timecourse and the HRF regression based response estimation when the underlying neural population undergoes real adaptation (226).

"To further understand whether the time course of our task could lead to artifacts, we also simulated responses to our task using tuned voxels that were modeled after the task sequence and estimated HRFs observed in our experiment (see supplementary modeling section, Figure S7). These simulations show that repulsive biases like the ones we observed with both our time course and deconvolution-based decoders are only possible when the underlying tuning of voxels is adapted by past stimuli/responses."

Finally, we did inspect orientation-selective HRFs by binning trials into 6 orientation bins. For each voxel, we compared their response to their preferred orientation (parameterized response with largest maximum value) to their non-preferred orientation (opposite orientation bin to preferred). These are presented in the figure below with the average preferred (black) and anti-preferred (red) HRFs for each ROI. We additionally tried to scale the anti-preferred responses to match the preferred response using an amplitude and constant term using linear regression (dotted teal line). We found that responses were approximately linearly scaled versions of each other, but that in areas beyond V1 the undershoot was typically less than expected for the preferred orientation. That said, we observed repulsive biases in all areas that we measured in the study, so the small changes in shape in some areas – and not in others – cannot account for all of the observed effects.

Neuroscience Graduate Program and Department of Psychology
University of California San Diego • 9500 Gilman Drive #0109 • La Jolla, California 92093
858-246-1340 • tsheehan@ucsd.edu

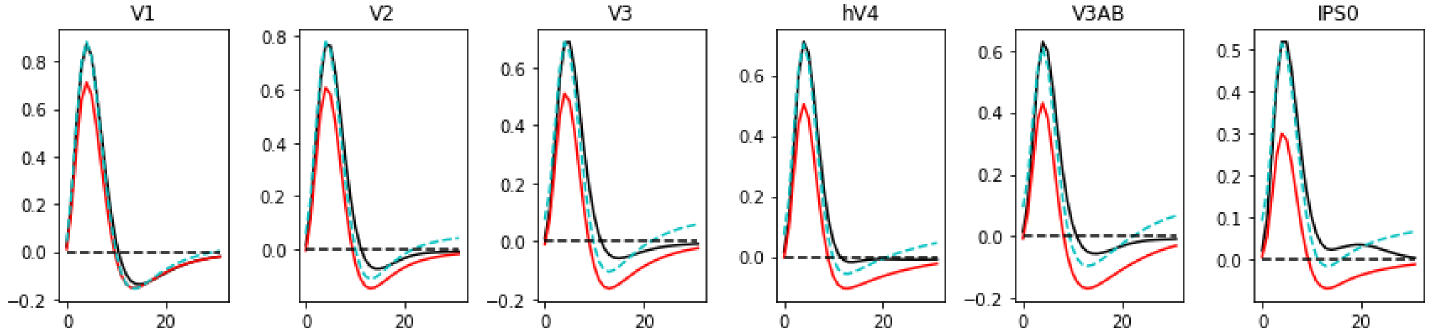

To directly assess any potential impact on decoding, we used these orientation specific HRFs to model BOLD responses is a similar manner to our deconvolution model (Figure S7). For each simulated voxel, we used the 6 HRFs estimated for a random voxel and rotated them such that the ‘preferred’ HRF aligned with that simulated voxel’s preferred orientation. These HRFs were convolved with the same amplitude stick functions as our BOLD simulation so the response on each trial was dictated by both the smoothly varying amplitude relating to that simulated voxels tuning curve and the binned HRF corresponding to that orientation range. The resulting curves (shown below for V1, matching caption of figure S7) show that there was no statistically significant bias found following the deconvolution process and any modulations were much smaller magnitude than the biases uncovered in our study. Along with the biases found by the time course decoder, these results along with those presented in the supplement (S7) converge to argue that the repulsive biases observed in our study or driven by adaptation in the underlying neural population.

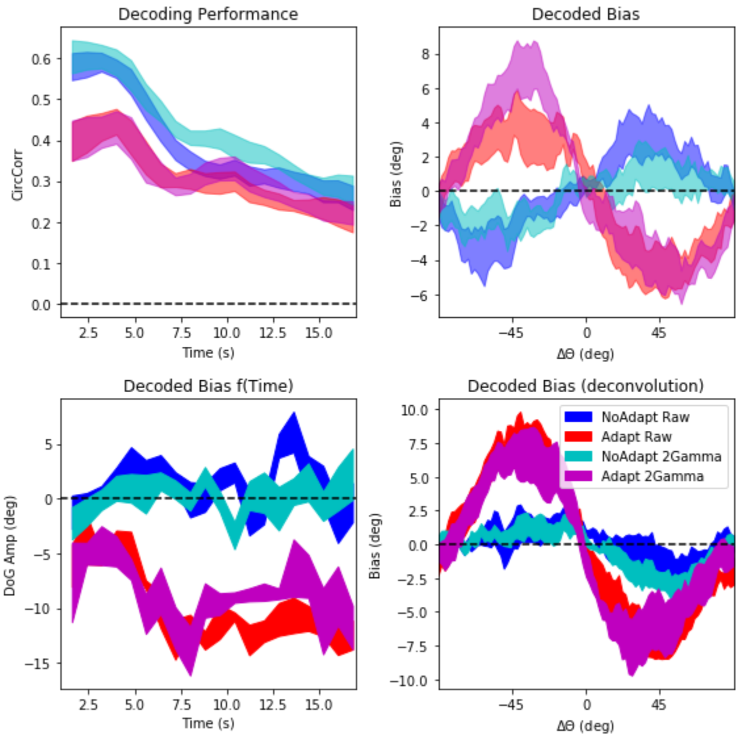

2. The analysis of the Stringer et al (2019) paper very nicely supports the conclusions of the authors. One detail (or analysis) that would help bolster this would be to ensure the reader that the statistics of the sequencing of stimulus events themselves were such that the repulsive biases were truly the result of neural processes, and not an accidental result of orientation sequences that have some non-randomness to them

Neuroscience Graduate Program and Department of Psychology
University of California San Diego • 9500 Gilman Drive #0109 • La Jolla, California 92093
858-246-1340 • tsheehan@ucsd.edu

that could have given rise to the observed effects. I imagine it's not a huge concern, but some more detail about those stimuli and the sequencing would be important here.

While the trial sequences seem to be truly random in this task, the timing of the trials and methods used to preprocess the data do seem to introduce artifacts into the analysis. As noted at the beginning of our response to the reviews, we have opted to remove this analysis from our paper due to this artifact.

3. The model that the authors put forth is interesting, but some aspects of it are puzzling and could use a bit more substantiating or caveats. In particular, I found myself puzzling over what a 'bases aware or hyper-aware' schema would look like, and how that would be possibly implemented. In particular, were these plausible scenarios? It would seem the "aware" class of models involve the system possessing a population that maintains a *veridical representation*, and can choose to compare it to the affected representation? I acknowledge this is just a model, but it seemed to me a bit strange for there to be a scenario in which this were implemented in the brain. I think the authors could do a better job substantiating these hypothetical alternative models, backing the possibilities up a little with more empirical precedent.

The 'aware' models do not necessarily require a separate representation of the veridical orientation, and we agree that such a schema would seem untethered to existing data and overly complex. Instead such a readout could simply require access to both the current and previous stimulus representations and could integrate this information. We present some additional evidence for the presence of this history information including a recent study demonstrating that prefrontal cortex can multiplex different items in a stimulus sequence by storing them in orthogonal codes (Xie et al., 2022). We additionally expand our discussion of a study which eliminated history biases by suppressing activity in mouse parietal cortex that represented sensory history (426).

"We did not explicitly define how awareness of adaptation is implemented, **but it is clear that both attention to and conscious awareness of the previous stimulus are necessary for serial dependence to occur (Fischer and Whitney, 2014; Kim et al., 2020).** This is consistent with our model, and it suggests that some representation of information about stimulus history should be a minimum requirement for an aware decoding scheme. The identity of the previous stimulus for spatial position and angle has been shown to be decodable from the spiking activity of single units in the frontal eye field (FEF) and large-scale activity patterns in human EEG **and MEG** (Papadimitriou et al., 2016; **Fornaciai and Park, 2018**; Bae and Luck, 2019; **Bae, 2021; Hajonides et al., 2021**). We additionally demonstrate that information about the previous trial is encoded in patterns of fMRI activity in human visual cortex (Figure 2F), **but not in a sensory-like code (Figure S4AB).** These signals could potentially be represented concurrently with representations of the current stimulus in the same populations of sensory neurons **but in orthogonal codes analogous to what has been found for sequentially encoded items in primate prefrontal cortex and human EEG (Wan et al., 2020; Xie et al., 2022)**. An alternate account holds that representations of stimulus history are maintained outside of early visual areas, consistent with findings from mouse parietal and **primate prefrontal cortex** (Akrami et al., 2018; **Barbosa et al., 2020**). This anatomical segregation could disambiguate incoming sensory drive from representations of stimulus history. **Critically, optogenetically suppressing non-sensory representations of stimulus history eliminated history effects, thus providing strong support for some form of an aware readout mechanism (Akrami et al., 2018)."**

Neuroscience Graduate Program and Department of Psychology
University of California San Diego • 9500 Gilman Drive #0109 • La Jolla, California 92093
858-246-1340 • tsheehan@ucsd.edu

UC San Diego

4. The 'awareness' component of this study, in particular its modeling component, should reference a recent paper by Kim, Burr, Cicchini & Alais (2020) that found that serial dependence did not emerge when stimuli were suppressed under binocular rivalry. This supports the notion put forth by the authors that this effect transpires later in the perceptual processing stream.

We now include behavioral studies in our discussion of the role of awareness of stimulus history in sensory processing including Kim et al., 2020. For example, on page XX, paragraph XX:

"We did not explicitly define how awareness of adaptation is implemented, **but it is clear that both attention to and conscious awareness of the previous stimulus are necessary for serial dependence to occur (Fischer and Whitney, 2014; Kim et al., 2020).**"

Regards,

Timothy Sheehan

Timothy C. Sheehan & John T. Serences, Ph.D.

Neuroscience Graduate Program and Department of Psychology
University of California San Diego • 9500 Gilman Drive #0109 • La Jolla, California 92093
858-246-1340 • tsheehan@ucsd.edu
