# Supplementary material for: Attractive serial dependence overcomes repulsive neuronal adaptation
Source: PLoS Biol. 2022 Sep 6;20(9):e3001711. doi: 10.1371/journal.pbio.3001711 (PMC9447932; doi:10.1371/journal.pbio.3001711)
Supplement: Attachment — Submitted filename: ReviewerResponseFinal.docx [file pbio.3001711.s011.docx]

Reviewer 1:

1. "The identity of the previous stimulus for spatial position and angle has been shown to be decodable from the spiking activity of single units in the frontal eye field (FEF)"

Here PPC in rats (Akrami at al) and PFC in monkeys (Barbosa et al) are other instances of previous stimulus decodability from spiking activity.

Line 430: Great point on the relevance of these additional studies. We now include a note on PPC decodability and cite Akrami et al.. We do not include Barbosa et al. on this particular point as they do not show decodability once a separate stimulus has been presented, only leading up to its onset. Thus it seems the activity patterns in PFC may be representing a phenomenon distinct from those shown in these other studies recoding from sensory and parietal areas.

“The identity of the previous stimulus for spatial position and angle has been shown to be decodable from the spiking activity of single units in the frontal eye field (FEF) and posterior parietal cortex (PPC) as well as large-scale activity patterns in human EEG and MEG (Papadimitriou et al., 2016; Akrami et al., 2018; Fornaciai and Park, 2018; Bae and Luck, 2019; Bae, 2021; Hajonides et al., 2021).”

2. "It is curious that unlike some spatial working memory studies (Papadimitriou et al., 2015; Bliss et al., 2017; Stein et al., 2020), we did not find that behavioral biases increased with delay time. One possible explanation is that this phenomenon is actually unique to spatial working memory due to either a more consistent increase in sensory uncertainty of spatial location due to eye movements or a separate mechanism of memory maintenance that becomes more susceptible to proactive interference relative to orientation memories."

I appreciate that the authors now fully acknowledge the discrepancy, but I don't think that different experimental modalities explain the differences. We have seen this effect in other modalities (unpublished), but see Fritsche (2017) for a very similar task. Finally, there is even recent theoretical arguments for this effect: <https://www.biorxiv.org/content/biorxiv/early/2022/03/02/2022.02.28.482269.full.pdf>

This is an interesting detail to know. We note that the Fritsche 2017 design as well as all spatial WM designs have the stimulus presented in the periphery. We now include an alternative account of foveal (and set size 1) representations potentially being less vulnerable to degradation.

“Separately, as our stimuli were presented at the fovea (unlike spatial paradigms) they are encoded by a larger population of sensory neurons and thus may be less susceptible to degradation across time.

The included reference suggesting a rate-distortion framing is interesting (and an area we are actively exploring) but we think beyond the scope of the current manuscript.

3. I find it a bit surprising that the new finding "decoding using sensory localizer data was consistently at chance during N+1 trial suggesting information relating to past stimulus is not stored in a sensory code." did not make it to the main manuscript. This is directly related with the title of the manuscript and claims raised in the abstract, so I think it should be giving its deserved attention in the main manuscript.

This is a fair point. We now include visually reported t-tests for both of these panels (Figure S4 A-B). When decoding using the sensory code, we do note that V2 is reliably above chance but not by enough to survive a multiple comparisons correction for all the ROIs we are examining. We now reference this finding in the text.

“Notably, we were generally unable to decode the identity of the previous stimulus using a decoder trained on a localizer task suggesting representations of past trial stimuli are not in a ‘sensory code’ (Figure S4B). Alternatively, the lack of attention to the stimulus in the sensory localizer task might lower SNR or alter the code in a manner that impairs generalizability.”

4. About my initial comment on 183 that you said it would be circular: I might be missing something, but I don't see why.

What you do now is plotting (prev_stimulus - curr_stimulus)  vs (pred_curr_stimulus - curr_stimulus).

What I was curious about was (pred_curr_stimulus - curr_stimulus) vs (pred_prev_stimulus - curr_stimulus) or vs (pred_prev_stimulus - pred_curr_stimulus).

pred = predicted from neural activity

Do you think this would be circular?

In any case, this is mostly my own curiosity, rather a requirement for acceptance.

Thanks for clarifying. Now that we understand, the proposed analysis does have some merit as one might expect that neural responses to be better explained by adaptation to previous neural activity/representations than past stimuli themselves. However, it becomes very challenging to interpret or to decide whether one should not then go further and sort trials by the difference between neural representations between consecutive trials (eg. x-axis: pred_prev_stimulus - pred_curr_stimulus). We prefer to condition our analysis on the only known variable defined by the experimenter to avoid potential confounds when sorting trials based on responses to stimuli (which are themselves biased). For example, Pascucci et al., demonstrated that sorting based on prior responses (instead of the stimuli which were constructed to be independent) can lead to artifactual “N+1” effects where future events appear to influence the current response distribution. This occurs because if there are biases in behavioral response patterns (e.g. cardinal biases, etc), then conditioning on a biased response will propagate these biases into future responses (neural or behavioral; eg. see examples of ‘spurious’ serial dependence in Pascucci et al., 2019, Figure 2F). Thus, while we agree completely that these kinds of analyses could be very informative, we explicitly opt to condition on known - and uniformly distributed - variables to align our work with previous studies of serial dependence and sensory adaptation.
